# Supplementary material for: Determinants of COVID-19 vaccine hesitancy and uptake in sub-Saharan Africa: a scoping review
Source: BMJ Open. 2022 Nov 17;12(11):e066615. doi: 10.1136/bmjopen-2022-066615 (PMC9676416; doi:10.1136/bmjopen-2022-066615)
Supplement: Supplementary data [file bmjopen-2022-066615supp001.pdf]

Supplementary file 1

The final search strategy was performed in PubMed, Scopus, Web of Science, Cochrane, Academic Search Premier, MEDLINE, Cumulative Index to Nursing and Allied Health Literature (CINAHL), Health Source Nursing, Africa Wide and APA PsychInfo on March 9, 2022, with the following search terms, where appropriate. We did not use any filters or limits in the search strategy to maximize the articles available to us.

COVID-19 Vaccines OR COVID-19 OR Coronavirus [Mesh]

"COVID-19" OR "coronavirus 2019" OR "SARS-CoV-2″ OR "SARS-2″ OR "severe acute respiratory syndrome coronavirus 2"

Vaccination Refusal [Mesh]

vaccination hesitancy OR vaccine hesitancy OR vaccine refusal OR vaccination refusal OR vaccine access OR access

Africa South of the Sahara [MeSH]

“Angola” OR “Benin” OR “Botswana” OR "Burkina Faso" OR “Burundi” OR “Cabo Verde” OR “Cameroon” OR “Cameroun” OR "Canary Islands" OR "Cape Verde" OR “Central Africa” OR "Central African Republic" OR “Chad” OR “Comoros” OR “Congo” OR "Cote d'Ivoire" OR "Democratic Republic of Congo" OR “Djibouti” OR “Eastern Africa” OR “Eritrea” OR “eSwatini” OR “Ethiopia” OR “Gabon” OR “Gambia” OR “Ghana” OR “Guinea” OR “Guinea-Bissau” OR "Ivory Coast" OR “Jamahiriya” OR “Kenya” OR “Lesotho” OR “Liberia” OR “Madagascar” OR “Malawi” OR “Mali” OR “Mauritania” OR “Mauritius” OR “Mayotte” OR “Mozambique” OR “Namibia” OR “Niger” OR “Nigeria” OR “Principe” OR “Reunion” OR “Rwanda” OR “Sao Tome” OR “Senegal” OR “Seychelles” OR “Sierra Leone” OR “Saint Helena” OR “Somalia” OR “St Helena” OR “South Africa” OR “Southern Africa” OR “Sudan” OR “Swaziland” OR “Tanzania” OR “Togo” OR “Uganda” OR “Western Africa” OR “Western Sahara” OR “Zaire” OR “Zambia” OR “Zimbabwe”
