# Supplementary material for: Determinants of COVID-19 vaccine hesitancy and uptake in sub-Saharan Africa: a scoping review
Source: BMJ Open. 2022 Nov 17;12(11):e066615. doi: 10.1136/bmjopen-2022-066615 (PMC9676416; doi:10.1136/bmjopen-2022-066615)
Supplement: Supplementary data [file bmjopen-2022-066615supp002.pdf]

Year	Author(s)	Title	Journal	Abstract
2021	Á F. L. de Sousa, J. R. B. Teixeira, I. Lua, F. O. Souza, A. J. F. Ferreira, G. Schneider, H. E. F. de Carvalho, L. B. de Oliveira, S. V. M. A. Lima, A. R. de Sousa, T. M. E. de Araújo, E. L. S. Camargo, M. O. B. Oriá, I. Craveiro, T. M. de Araújo, I. A. C. Mendes, C. A. A. Ventura, I. Sousa, R. M. de Oliveira, M. Simão and I. Fronteira	Determinants of COVID-19 vaccine hesitancy in portuguese-speaking countries: A structural equations modeling approach	Vaccines	COVID-19 vaccine hesitancy (VH) has caused concerns due to the possible fluctuations that may occur directly impacting the control of the pandemic. In this study, we aimed to estimate the prevalence and factors associated with COVID-19 VH in Portuguese-speaking countries. We developed a web survey (N:6,843) using an online, structured, and validated questionnaire. We used Measurement Models, Exploratory Factor Analysis, Exploratory Structural Equation Models, and Confirmatory Factor Analysis for the data analysis. The overall prevalence of COVID-19 VH in Portuguese-speaking countries was 21.1%. showed a statistically significant direct effect for VH: vaccine-related conspiracy beliefs (VB) (β = 0.886), perceived stress (PS) (β = 0.313), COVID-19 Misinformation (MIS) (β = 0.259) and individual responses to COVID-19 (CIR) (β = −0.122). The effect of MIS and CIR for VH was greater among men and of PS and VB among women; the effect of PS was greater among the youngest and of VB and CIR among the oldest. No discrepant differences were identified in the analyzed education strata. In conclusion, we found that conspiracy beliefs related to the vaccine strongly influence the decision to hesitate (not to take or to delay the vaccine). Specific characteristics related to gender, age group, social and cognitive vulnerabilities, added to the knowledge acquired, poorly substantiated and/or misrepresented about the COVID-19 vaccine, need to be considered in the planning of vaccination campaigns. It is necessary to respond in a timely, fast, and accurate manner to the challenges posed by vaccine hesitancy. © 2021 by the authors. Licensee MDPI, Basel, Switzerland.
2021	A. A. Shamshirsaz, K. Hessami, S. Morain, Y. Afshar, A. A. Nassr, S. E. Arian, N. M. Asl and K. Aagaard	Intention to Receive COVID-19 Vaccine during Pregnancy: A Systematic Review and Meta-analysis	American Journal of Perinatology	Objective This meta-analysis aimed to assess the level of intent to receive coronavirus disease 2019 (COVID-19) vaccination and demographical factors influencing vaccine uptake among pregnant individuals. Study Design PubMed, Scopus, and archive/pre-print servers were searched up to May 22nd, 2021. Cross sectional surveys reporting the percentage of the pregnant individuals intending to get a COVID-19 vaccine were considered eligible for meta-analysis. This review was registered with PROSPERO (CRD42021254484). The primary outcome was to estimate the prevalence of COVID-19 vaccination intent among pregnant population. The secondary outcome was to evaluate the factors influencing the intention for vaccination. Results Twelve studies sourcing data of 16,926 individuals who were identified as pregnant were eligible. The estimated intention for the receipt of COVID-19 vaccine among women who were pregnant was 47% (95% CI: 38-57%), with the lowest prevalence in Africa 19% (95% CI: 17-21%) and the highest in Oceania 48.0% (95% CI: 44.0-51.0%). Uptake of other vaccines (influenza and/or TdaP) during pregnancy was associated with higher rate of intent to receive the COVID-19 vaccine (OR = 3.03; 95% CI: 1.37-6.73; p = 0.006). Conclusion The intent to receive COVID-19 vaccine is relatively low among women who are pregnant and substantially varies based on the country of residence. In our meta-analysis, intent of women who were pregnant to receive the COVID-19 vaccine was significantly associated with the history of receiving influenza or TdaP vaccine during pregnancy. Given that in every country only a minority of gravidae have received the COVID-19 vaccine, despite known risks of maternal morbidity and mortality with no evidence of risks of vaccination, it highlights the importance of revised approaches at shared decision making and focused public health messaging by national and international advisories. Key Points The estimated global intention for COVID-19 vaccination among pregnant women was 47%. The lowest intention was in Africa and the highest in Oceania. These findings highlight the importance of public health messaging by by different agencies. © 2021 Georg Thieme Verlag. All rights reserved.

Year	Author(s)	Title	Journal	Abstract
2021	A. D. Wake	The Acceptance Rate Toward COVID-19 Vaccine in Africa: A Systematic Review and Meta-analysis	Global Pediatric Health	Background: The Coronavirus Disease 2019 (COVID-19) pandemic remains serious public issue. COVID-19 vaccine is a vital strategy to prevent this critical pandemic. However, unwillingness to take this vaccine are key barriers to manage the COVID-19 pandemic. The control of this pandemic will depend principally on the people acceptance of COVID-19 vaccine. Therefore, this systematic review and meta-analysis was intended to determine the acceptance rate toward COVID-19 vaccine in Africa. Methods: African Journals OnLine, PubMed, Cochrane Review, HINARI, EMBASE, Google Scholar, Web of Science, and Scopus were used to retrieve related articles. The Preferred Reporting Items for Systematic Review and Meta-Analysis (PRISMA) guidelines were used for this study. Random-effect model, a funnel plot, Egger's test, I-2 statistic, subgroup analysis was done. The study was performed by using a STATA version 11 statistical software. Results: A total of 22 studies with 33,912 study participants were included in this systematic review and meta-analysis. From this finding, the pooled prevalence of acceptance toward COVID-19 vaccine among adults in Africa was 48.93% (95% CI: [39.49, 58.37]). The subgroup analysis revealed that the pooled prevalence of COVID-19 vaccine acceptance among adults in Africa was highest (66.03%, 95% CI [62.84, 69.22]) in Southern Africa, and Lowest (24.28%, 95% CI [3.26, 45.30]) in Northern Africa. Conclusion: This study showed that the estimate of the pooled prevalence of acceptance toward COVID-19 vaccine among adults in Africa was very low. All concerned bodies should be actively involved to improve the acceptance rate of COVID-19 vaccine.
2022	A. I. Al-Mustapha, M. I. Abubakar, M. Oyewo, R. E. Esighetti, O. A. Ogundijo, L. D. Bolanle, O. E. Fakayode, A. S. Olugbon, M. Oguntoye and N. Elelu	Socio-Demographic Characteristics of COVID-19 Vaccine Recipients in Kwara State, North Central Nigeria	Frontiers in Public Health	Understanding key socio-demographic variables of 2019 coronavirus disease (COVID-19) vaccine recipients is crucial to improving its acceptance and Nigeria's COVID-19 control strategy. The survey was conducted as a non-probability cross-sectional survey of 2,936 COVID-19 vaccine recipients in Kwara State. Our findings revealed that 74% (n = 2,161) of the vaccine recipients were older than 40 years. Forty percent (n = 1,180) of the vaccine recipients earned a monthly income >100,000 Naira (equivalent to US $200). Most of the vaccine recipients (64%, n = 1,880) had tertiary education, while 15% (n = 440) of them had no formal education. Almost half of the recipients (47%, n = 1,262) were government employees and 28.8% (n = 846) of them had health-related backgrounds. Only 17% (n = 499) of the vaccine recipients have been screened for the severe acute respiratory syndrome coronavirus 2 (SARS-CoV-2), of which 21% (n = 105/499) of them were tested positive. Only 47% (n = 1,378) had been fully immunized. The prevalence of confirmed COVID-19 cases among COVID-19 vaccine recipients in Kwara State was 3.6% (n = 105/2,936). The most recurrent adverse events following immunization (AEFIs) among vaccine recipients were fever (14%, n = 411), pain at injection site (47%, n = 1,409), headache (19%, n = 558), and body weakness (9%, n = 264). The need to protect themselves from the deadly virus was the main reason that prompted people to voluntarily accept the COVID-19 vaccine. There is a high level of COVID-19 vaccine acceptance among respondents across all social classes including those with no formal education, those with very low monthly income (< US $2 per day), and in untested population. Hence, vaccine donors should prioritize equitable distribution to Low-and-Middle-income Countries (LMICs) such as Nigeria, and health authorities should improve vaccine advocacy to focus on vaccine safety and efficacy. Copyright © 2022 Al-Mustapha, Abubakar, Oyewo, Esighetti, Ogundijo, Bolanle, Fakayode, Olugbon, Oguntoye and Elelu.

Year	Author(s)	Title	Journal	Abstract
2021	A. Mose and A. Yeshaneh	COVID-19 vaccine acceptance and its associated factors among pregnant women attending antenatal care clinic in southwest ethiopia: Institutional-based cross-sectional study	International Journal of General Medicine	Background: COVID-19 vaccination is a safe and effective approach to control the pandemic and to prevent its associated morbidity and mortality. To our knowledge, there is no study conducted to assess the prevalence of COVID-19 vaccine acceptance among pregnant women in Ethiopia. Therefore, the main objective of this study was to assess the prevalence of COVID-19 vaccine acceptance and its associated factors among pregnant women attending antenatal care clinic in Southwest Ethiopia. Methods: An institution-based cross-sectional study was employed from January 1 up to 30, 2021. A systematic random sampling technique was used to select 396 study participants. A structured and face-to-face interview was used to collect data. Data were entered into Epi-data version 4.2.0 and exported to SPSS version 23 for analysis. Bivariate and multivariate analyses were used to identify factors associated with COVID-19 vaccine acceptance. P values <0.05 result were considered as a statistically significant association. Results: The COVID-19 vaccine acceptance was found to be 70.7% (95% CI, 66.7%– 74.7%). Maternal age (34–41) years [AOR=1.464, (95% CI; 1.218–5.129)], primary maternal educational status [AOR=3.476, (95% CI; 1.520–7.947), good knowledge [AOR=5.946, (95% CI; 3.147–7.065)], and good practice [AOR =9.15, (95% CI; 8.734–12.189)] of pregnant women towards COVID-19 and its preventive measures were factors associated with COVID-19 vaccine acceptance. Conclusion: COVID-19 vaccine acceptance was found to be 70.9%. Maternal age (34–41) years, primary maternal educational status, good knowledge, and good practice of pregnant women towards COVID-19 and its preventive measures were factors associated with COVID-19 vaccine acceptance. Health care workers should provide health education to pregnant women to increase their knowledge about the diseases and disseminate leaflets regarding COVID-19 preventive measures. Moreover, before initiation of COVID-19 vaccine administration to pregnant women they must promote the safety and effectiveness of COVID-19 vaccine. © 2021 Mose and Yeshaneh.
2021	A. T. Chinawa, J. M. Chinawa, E. N. Ossai, N. Obinna, V. Onukwuli, A. E. Aronu and C. P. Manyike	Maternal level of awareness and predictors of willingness to vaccinate children against COVID 19; A multi-center study	Human Vaccines and Immunotherapeutics	Background: Several controversies surround mothers' willingness to vaccinate against the COVID-19 pandemic especially when mortality is not frequently reported in children. Objectives: This study aimed to ascertain the willingness of mothers of children attending two institutions in Southeast Nigeria to accept the COVID-19 vaccine and factors that may be associated with their choices. Methodology: This was a cross-sectional study carried out among 577 mothers who presented with their children in two tertiary health institutions in southeast Nigeria. Results: Majority of the respondents (93.9%) were aware of the COVID-19 vaccine. Majority of the respondents, 89.4%, noted that children were not in high priority groups for COVID-19 vaccination in Nigeria. Only 6.9% of the respondents intend to receive the COVID-19 vaccination. Also, a minor proportion of the respondents (4.9%) were willing to vaccinate their children with the COVID-19 vaccine. The odds of receiving the Covid-19 vaccine were four times greater in those who believed that they could be infected than in those who believed that they could not be infected (AOR = 4.0. 95% CI:1.8–8.7). The odds of receiving the Covid-19 vaccine were six times greater in those who were aware of someone that died from COVID-19 than in those who did not know anyone who died from COVID-19 (AOR = 5.7, 95% CI: 2.1–15.8). Conclusion: A high level of awareness but low acceptance level for COVID-19 vaccination for mothers and their children was noted. Socioeconomic class, maternal age, and level of education did not influence the willingness of the mother to receive COVID vaccination. Having a belief of possibility of infection with the COVID-19 as well as being aware of someone who died from the disease were important positive variables that could predict vaccine acceptance from this study. © 2021 Taylor & Francis Group, LLC.

Year	Author(s)	Title	Journal	Abstract
2021	Abayomi Samuel Oyekale	Compliance Indicators of COVID-19 Prevention and Vaccines Hesitancy in Kenya: A Random-Effects Endogenous Probit Model	Vaccines	Vaccine hesitancy remains a major public health concern in the effort towards addressing the COVID-19 pandemic. This study analyzed the effects of indicators of compliance with preventive practices on the willingness to take COVID-19 vaccines in Kenya. The data were from the COVID-19 Rapid Response Phone Surveys conducted between January and June 2021 during the fourth and fifth waves. The data were analyzed with the random-effects endogenous Probit regression model, with estimated parameters tested for robustness and stability. The results showed that willingness to take vaccines increased between the fourth and fifth waves. Compliance with many of the preventive practices also improved, although the utilizations of immune system-promoting practices were very low. The panel Probit regression results showed that compliance indicators were truly endogenous and there was existence of random effects. Immune system-boosting and contact-prevention indicators significantly increased and decreased the willingness to take vaccines, respectively ($p < 0.01$). The experience of mental health disorders in the form of nervousness and hopelessness also significantly influenced vaccine hesitancy ($p < 0.10$). Willingness to take vaccines also significantly increased among older people and those with a formal education ($p < 0.01$). Different forms of association exist between vaccine hesitancy and the prevention compliance indicators. There is a need to properly sensitize the people to the need to complement compliance with COVID-19 contact-prevention indicators with vaccination. Addressing mental health disorders in the form of loneliness, nervousness, depression, hopelessness and anxiety should also become the focus of public health, while efforts to reduce vaccine hesitancy should focus on individuals without formal education, males and youths.
2021	AbdulAzeez A. Anjorin, Ismail A. Odetokun, Ajibola I. Abioye, Hager Elnadi, Mfon Valencia Umoren, Bamu F. Damaris, Joseph Eyedo, Haruna I. Umar, Jean B. Nyandwi, Mena M. Abdalla, Sodiq O. Tijani, Kwame S. Awiagah, Gbolahan A. Idowu, Sifeuh N. Achille Fabrice, Aala M. O. Maisara, Youssef Razouqi, Zuhal E. Mhgoob, Salim Parker, Osaretin E. Asowata and Ismail O. Adesanya	Will Africans take COVID-19 vaccination?	PLoS ONE	The economic and humanistic impact of COVID-19 pandemic is enormous globally. No definitive treatment exists, hence accelerated development and approval of COVID-19 vaccines, offers a unique opportunity for COVID-19 prevention and control. Vaccine hesitancy may limit the success of vaccine distribution in Africa, therefore we assessed the potentials for coronavirus vaccine hesitancy and its determinants among Africans. An online cross-sectional African-wide survey was administered in Arabic, English, and French languages. Questions on demographics, self-reported health status, vaccine literacy, knowledge and perception on vaccines, past experience, behavior, infection risk, willingness to receive and affordability of the SARS-COV-2 vaccine were asked. Data were subjected to descriptive and inferential statistics. A total of 5,416 individuals completed the survey. Approximately, 94% were residents of 34 African countries while the other Africans live in the Diaspora. Only 63% of all participants surveyed were willing to receive the COVID-19 vaccination as soon as possible and 79% were worried about its side effects. Thirty-nine percent expressed concerns of vaccine-associated infection. The odds of vaccine hesitancy was 0.28 (95% CI: 0.22, 0.30) among those who believed their risk of infection was very high, compared to those who believed otherwise. The odds of vaccine hesitancy was one-fifth (OR = 0.21, 95% CI: 0.16, 0.28) among those who believed their risk of falling sick was very high, compared to those who believed their risk of falling very sick was very low. The OR of vaccine hesitancy was 2.72 (95% CI: 2.24, 3.31) among those who have previously refused a vaccine for themselves or their child compared to counterparts with no self-reported history of vaccine hesitancy. Participants want the vaccines to be mandatory (40%), provided free of charge (78%) and distributed in homes and offices (44%). COVID-19 vaccine hesitancy is substantial among Africans based on perceived risk of coronavirus infection and past experiences. [ABSTRACT FROM AUTHOR]

Year	Author(s)	Title	Journal	Abstract
2021	Abiy Tadesse Angelo, Daniel Shiferaw Alemayehu and Aklilu Mamo Dachew	Health care workers intention to accept COVID-19 vaccine and associated factors in southwestern Ethiopia, 2021	PLOS ONE	Introduction Health care workers are the most affected part of the world population due to the COVID-19 pandemic. Countries prioritize vaccinating health workers against COVID-19 because of their susceptibility to the virus. However, the acceptability of the vaccine varies across populations. Thus, this study aimed to determine the health care worker's intentions to accept the COVID-19 vaccine and its associated factors in southwestern Ethiopia, 2021. Methods A facility-based cross-sectional study was conducted among health care workers in public hospitals in southwestern Ethiopia from March 15 to 28, 2021. A simple random sampling method was used to select 405 participants from each hospital. Data were collected using self-administered questionnaires. Descriptive statistics, such as frequency and percentage, were calculated. Multivariable logistic regression was also performed to identify factors associated with health care worker's intention to accept the COVID-19 vaccine. Statistically significant variables were selected based on p-values (<0.05) and the adjusted odds ratio was used to describe the strength of association with 95% confidence intervals. Result Among the respondents, 48.4% [95% CI: 38.6, 58.2] of health care workers intended to accept COVID-19. Intention to accept COVID-19 vaccination was significantly associated with physicians (AOR = 9.27, 95% CI: 1.27–27.32), professionals with a history of chronic illness (AOR = 4.07, 95% CI: 2.02–8.21), perceived degree of risk of COVID-19 infection (AOR = 4.63, 95% CI: 1.26–16.98), positive attitude toward COVID-19 prevention (AOR = 6.08, 95% CI: 3.39–10.91) and good preventive practices (AOR = 2.83, 95% CI: 1.58–5.08). Conclusion In this study, the intention of health care workers to accept the COVID-19 vaccine was low. Professional types, history of chronic illness, perceived degree of risk to COVID-19 infection, attitude toward COVID-19 and preventive practices were found to be factors for intention to accept COVID-19 vaccine in professionals. It is important to consider professional types, history of chronic illness, perceived degree of risk to COVID-19, attitude of professionals and preventive behaviors to improve the intention of professionals' vaccine acceptance.
2021	Agazhe Aemro, Nakachew Sewnet Amare, Belayneh Shetie, Basazinew Chekol and Mulugeta Wassie	Determinants of COVID-19 vaccine hesitancy among health care workers in Amhara region referral hospitals, Northwest Ethiopia: a cross-sectional study	Epidemiology & Infection	
2021	Andrea C Carcelen, Christine Prosperi, Simon Mutembo, Gershom Chongwe, Francis D Mwansa, Phillimon Ndubani, Edgar Simulundu, Innocent Chilumba, Gloria Musukwa and Phil Thuma	COVID-19 vaccine hesitancy in Zambia: A glimpse at the possible challenges ahead for COVID-19 vaccination rollout in sub-Saharan Africa	Human Vaccines & Immunotherapeutics	

Year	Author(s)	Title	Journal	Abstract
2021	Andrew Marvin Kanyike, Ronald Olum, Jonathan Kajjimu, Daniel Ojilong, Gabriel Madut Akech, Dianah Rhoda Nassozi, Drake Agira, Nicholas Kisaakye Wamala, Asaph Asiimwe, Dissan Matovu, Ann Babra Nakimuli, Musilim Lyavala, Patricia Kulwenza, Joshua Kiwumulo and Felix Bongomin	Acceptance of the coronavirus disease-2019 vaccine among medical students in Uganda	Tropical Medicine & Health	Background: COVID-19 is still a major global threat for which vaccination remains the ultimate solution. Uganda reported 40,751 cases and 335 deaths as of 9 April 2021 and started its vaccination program among priority groups like health workers, teachers, those with chronic diseases among others in early March 2021. Unanimous uptake of the COVID-19 vaccine is required to subsequently avert its spread; therefore, we assessed COVID-19 vaccine acceptability, hesitancy, and associated factors among medical students in Uganda. Methods: This study employed an online descriptive cross-sectional survey among medical students across 10 medical schools in Uganda. A structured questionnaire via Google Form was conveniently sent to eligible participants via WhatsApp. Each medical school had a coordinator who consistently shared the data tool in the WhatsApp groups. Chi-square or Fisher's exact test, and logistic regression were used to assess the association between vaccine acceptability with demographics, COVID-19 risk perception, and vaccine hesitancy. Results: We surveyed 600 medical students, 377 (62.8%) were male. COVID-19 vaccine acceptability was 37.3% and vaccine hesitancy 30.7%. Factors associated with vaccine acceptability were being male (adjusted odds ratio (aOR) = 1.9, 95% CI 1.3-2.9, p=0.001) and being single (aOR= 2.1, 95% CI 1.1-3.9, p=0.022). Very high (aOR= 3.5, 95% CI 1.7-6.9, p<0.001) or moderate (aOR =2.2, 95% CI 1.2-4.1, p=0.008) perceived risk of getting COVID-19 in the future, receiving any vaccine in the past 5 years (aOR= 1.6, 95% CI 1.1-2.5, p=0.017), and COVID-19 vaccine hesitancy (aOR 0.6, 95% CI 0.4-0.9, p=0.036). Conclusions: This study revealed low levels of acceptance towards the COVID-19 vaccine among medical students, low self-perceived risks of COVID-19, and many had relied on social media that provided them with negative information. This poses an evident risk on the battle towards COVID-19 in the future especially when these future health professions are expected to be influencing decisions of the general public towards the same. [ABSTRACT FROM AUTHOR]
2022	Ayenew Mose, Kassahun Haile and Abebe Timerga	COVID-19 vaccine hesitancy among medical and health science students attending Wolkite University in Ethiopia	PLoS ONE	Background: Medical and health science students are among the frontline health care workers who are at high risk of acquiring COVID-19 infection during their clinical attachments and future career. As health care providers, they are expected to promote and administer the COVID-19 vaccine and counsel vaccine-hesitant patients. It is, therefore, imperative to assess COVID-19 vaccine hesitancy among medical and health science students. Thus, this study aimed to assess COVID-19 vaccine hesitancy and its associated factors among medical and health science students of Wolkite University. Method: An institutional-based cross-sectional study design was conducted among 420 medical and health science students attending Wolkite University from March 1 to 30, 2021. Simple random sampling technique was used to select study participants. Self-administered and structured questionnaires were used to collect data. Data were entered into Epi-Data version 4.2.0 and exported to SPSS version 23 software package for further analysis. Bivariable and multivariable analysis was used to identify associated factors. P values <0.05 result were considered as a statistically significant association. Results: The level of COVID-19 vaccine hesitancy was 41.2% (95% CI; 35.2%-50.4%). Student age ≤23 years were 1.9 times more likely vaccine hesitant [aOR = 1.94, 95% CI; 1.14–3.28], being female were 1.7 times more likely vaccine hesitant [aOR = 1.76, 95% CI; 1.14–2.72], resided in rural area were 1.6 times more likely vaccine hesitant [aOR = 1.63, 95% CI; 1.06–2.49], source of information from social media were 2.7 times more likely vaccine hesitant [aOR = 2.68, 95% CI; 1.58–4.54], and good practice to COVID-19 mitigation measures were 47% less likely vaccine hesitant [aOR = 0.53, 95% CI; 0.34–0.83] compared to their counterpart. Conclusions: COVID-19 vaccine hesitancy is found to be high. Therefore, students are advised to receive COVID-19 vaccine information from government lead mass media (i.e. television and radio), increase awareness and adherence to COVID-19 mitigation measures is recommended. [ABSTRACT FROM AUTHOR]

Year	Author(s)	Title	Journal	Abstract
2021	B. O. Botwe, W. K. Antwi, J. A. Adusei, R. N. Mayeden, T. N. Akudjedu and S. D. Sule	COVID-19 vaccine hesitancy concerns: Findings from a Ghana clinical radiography workforce survey	Radiography (London, England : 1995)	Introduction: Vaccination is a key global strategy to mitigate the clinical impact of the COVID-19 virus. As part of local efforts to manage the outbreak, the government of Ghana announced its intention to vaccinate its population starting with essential and high-risk workers including radiographers. However, there were reports of hesitance to receiving the vaccine among the radiography workforce. This study was undertaken prior to the intended vaccination exercise to assess the willingness and concerns of radiographers to undergo the COVID-19 vaccination and to suggest recommendations to improve the vaccine uptake.; Methods: An ethically-approved online survey strategy was employed for this cross-sectional study conducted between 24th-28th February 2021. The survey employed quantitative questions and open text response options. Quantitative and open text responses were analysed using statistical and thematic analyses, respectively.; Results: There were 108 responses (response rate of 46.3%). The majority (n = 64, 59.3%) were willing to have the vaccine, however, some (n = 44, 40.7%) were not. The main reason behind their willingness to have the vaccine was its ability to reduce the spread of infections and lower mortality (n = 35, 54.7%). However, doubts about the vaccine's efficacy and side effects (n = 26, 56.8%), conspiracy theory concerns about its effects on the Ghanaian race (n = 4, 9.1%), and fertility concerns (n = 2, 4.5%) were some reasons for their hesitance to receive the vaccine. The open text commentary further revealed that the vaccine was thought of as a lifesaving medication, however, clinical safety concerns, lack of education/information and religious beliefs were affecting peoples' willingness to be vaccinated.; Conclusion: Our findings demonstrate the need for an urgent public health educational intervention to address the COVID-19 vaccine hesitancy concerns raised by radiographers to help increase the vaccine uptake.; Implication for Practice: The study provides pertinent information to improve COVID-19 vaccine uptake among radiographers to limit the spread of infections. (Copyright © 2021 The College of Radiographers. Published by Elsevier Ltd. All rights reserved.)
2021	B. T. Taye, F. K. Amogne, T. L. Demisse, M. S. Zerihun, T. M. Kitaw, A. E. Tiguh, M. S. Mihret and A. A. Kebede	Coronavirus disease 2019 vaccine acceptance and perceived barriers among university students in northeast Ethiopia: A cross-sectional study	Clin Epidemiol Glob Health	BACKGROUND: Universities are places where students live and study in close contact to each other. Nowadays, the foundations of this particular group have been affected significantly by the rapid spread of the coronavirus disease 2019. The severity of the COVID-19 pandemic has demanded the emergency use of COVID-19 vaccines. However, there is still limited evidence in COVID-19 vaccine acceptability and perceived barriers among some subgroups, including university students. This study aimed to assess vaccine acceptance, associated factors, and perceived barriers among university students, Ethiopia. METHODS: A cross-sectional study was conducted in January 2021 at Debre Berhan University among 423 students. The participants were selected using simple random sampling technique. A semi-structured, pretested, and self-administered questionnaire was used to collect the data. Multivariable logistic-regression model was fitted to identify factors associated with vaccine acceptance. An adjusted odds ratio with 95% confidence interval and its p-value of ≤0.05 was used to declare significant association. RESULTS: The proportion of the COVID-19 vaccine acceptance was 69.3% (95% CI: 65, 74). Being knowledgeable (AOR: 2.43, CI: 1.57, 3.77), being a health science student (AOR: 2.25, CI: 1.43, 3.54), and being in a family practicing COVID-19 prevention (AOR: 1.73, CI: 1.06, 2.81) were found to be factors associated with COVID-19 vaccine acceptance. CONCLUSION: Though, this study found a 69.3% acceptance of COVID-19 vaccine, there were noticeable perceived barriers and related factors in vaccine acceptance hesitancy. Thus, health education and communication regarding the vaccine are very crucial to alleviate the identified barriers.

Year	Author(s)	Title	Journal	Abstract
2021	Bewunetu Zewude and Tewodros Habtegiorgis	Willingness to Take COVID-19 Vaccine Among People Most at Risk of Exposure in Southern Ethiopia	Pragmatic and observational research	Background: Acceptance of a vaccine or hesitancy towards it have great public health implications as they partly determine the extent to which people are exposed to infections that could have otherwise been prevented. The present study examined the willingness of primary and secondary school teachers, bank employees, and university instructors in southern Ethiopia to take a Covid-19 vaccine and the factors associated with their willingness.; Methods: An institutional-based cross-sectional study design was used with a quantitative research approach. Primary data were gathered mainly through the use of a survey research method in which a self-administered questionnaire was distributed to randomly selected research participants in Wolaita Sodo town. Data analysis was conducted using statistical techniques, including percentages, frequency distributions, and logistic regression analysis.; Results: Research participants generally had a low (46.1%) willingness to take a COVID-19 vaccine. The main reason for most (37%) respondents' hesitancy to take the vaccine is found to be the concern over the safety and/or the side effects of the vaccine (37%), followed by doubt about the vaccine's effectiveness (20.7%), and lack of adequate information (12.7%). Moreover, 38.9% of survey participants revealed that they would like to take a COVID-19 vaccine other than AstraZeneca whereas 61.1% of respondents replied that they do not want to take any kind of COVID-19 vaccine. Furthermore, respondents' willingness to take a COVID-19 vaccine is significantly associated with attitude towards the vaccine (OR = 2.830; 95% CI = 1.834-4.368), belief that Covid-19 exists in the study area (OR = 0.221; 95% CI = 0.083-0.589), the perception that prevalence and death rate reports of the government are real (OR = 0.365; 95% CI = 0.197-0.676), status of chronic diseases (OR = 2.883; 95%CI = 1.039-7.999), and having a close relative/friend ever infected by COVID-19 (OR = 2.602; 95% CI = 1.117-6.063).; Conclusion: The findings of the research demonstrated that there is generally low willingness to take a COVID-19 vaccine among university instructors, bank employees, and primary and secondary school teachers in southern Ethiopia. Therefore, the federal ministry of health, Ethiopian food and drug controlling agency, the media, and all other concerned organizations should create increased awareness about the safety/side effects issues and the need to take the vaccine. (© 2021 Zewude and Habtegiorgis.)

Year	Author(s)	Title	Journal	Abstract
2022	C. Kassa Mekonnen, N. Gizaw Demissie, Z. Wako Beko, Y. Mulu Ferede and H. Kindie Abate	Intent to get vaccinated against COVID-19 pandemic and its associated factors among adults with a chronic medical condition	Int J Afr Nurs Sci	BACKGROUND: Vaccination intent is defined as the willingness to get vaccinated against a COVID-19 pandemic in a situation where the vaccine is available at no cost. Nevertheless, even with the availability of COVID-19 vaccines, some part of the public is not expected to get vaccinated, mainly due to a phenomenon known as vaccine hesitancy or lack of intention. Furthermore, there is little information available on the intention of people with chronic medical conditions about the COVID-19 vaccines in Ethiopia. OBJECTIVE: The study aimed to assess the intent to get vaccinated against COVID-19 and its associated factors among adults with a chronic medical condition. METHOD: An institutional-based cross-sectional study was conducted from February 15 to March 15, 2021. Simple random sampling was employed to get representative samples. Data were collected by using a structured questionnaire through face to face interviews. The data were entered by Epi Info version 7 and analyzed with SPSS version 20. The data were analyzed by using binary logistic regression. Those variables with a P-value of ≤ 0.05 were considered significantly associated with the outcome variable. RESULT: In this study, 423 study participants took part with a response rate of 100%. The mean age of the study participants was 50.07 (SD ± 13.7) with a range of 18-85 years. The intention to get vaccinated against the COVID-19 pandemic was 63.8% [95% CI (58.6-68.2)]. In the multivariable analysis the variables, retiring from the job was [AOR = 2.65, 95% CI (1.02-10.35)], having health insurance coverage [AOR = 1.38, 95%CI (1.04-3.65)], being in the high socio-demographic status [AOR = 1.67, 95%CI (1.01-2.78)], being confident with the Country's health care system [AOR = 2.00, 95%CI (1.15-3.49)], and having good knowledge about COVID-19 [AOR = 6.59, 95% CI (4.02-10.78)] were significant predictors of intent to get vaccinated against COVID-19 pandemic compared. CONCLUSION: The intention of getting vaccinated against the COVID-19 pandemic was relatively low. There has to be a great effort by the health caregivers as well as the government to increase vaccination intake, particularly for these priority groups.
2022	C. S. Wiysonge, S. M. Alobwede, P. D. C. Katoto, E. B. Kidzeru, E. N. Lumngwena, S. Cooper, R. Goliath, A. Jackson and M. S. Shey	COVID-19 vaccine acceptance and hesitancy among healthcare workers in South Africa	Expert Review of Vaccines	BACKGROUND We assessed willingness to accept vaccination against coronavirus disease 2019 (COVID-19) among healthcare workers(HCWs) at the start of South Africa’s vaccination roll-out. RESEARCH DESIGN AND METHODS We conducted a cross-sectional survey among HCWs in Cape Town in March-May 2021 and assessed predictors of vaccination intentions. RESULTS We recruited 395 participants; 64% women, 49% nurses, and 13% physicians. Of these, 233(59.0%) would accept and 163 (41.0%) were vaccine hesitant i.e. would either refuse or were unsure whether they would accept COVID-19 vaccination. People who did not trust that COVID-19 vaccines are effective were the most hesitant (p = 0.038). Older participants and physicians were more likely to accept vaccination than younger participants (p < 0.01) and other HCWs (p = 0.042) respectively. Other predictors of vaccine acceptance were trust that vaccines are compatible with religion (p < 0.001), consideration of benefits and risks of vaccination (p < 0.001), willingness to be vaccinated to protect others (p < 0.001), and viewing vaccination as a collective action for COVID-19 control (p = 0.029). CONCLUSIONS COVID-19 vaccine hesitancy is high among HCWs in Cape Town. Reducing this would require trust-building interventions, including tailored education.
2022	Chizoba Wonodi, Chisom Obi-Jeff, Funmilayo Adewumi, Somto Chloe Keluo-Udeke, Rachel Gur-Arie, Carleigh Krubiner, Elana Felice Jaffe, Tobi Bamiduro, Ruth Karron and Ruth Faden	Conspiracy theories and misinformation about COVID-19 in Nigeria: Implications for vaccine demand generation communications	Vaccine	

Year	Author(s)	Title	Journal	Abstract
2022	Christoph Strupat, Zemzem Shigute, Arjun S. Bedi and Matthias Rieger	Willingness to take COVID-19 vaccination in low-income countries: Evidence from Ethiopia	PLoS ONE	Background: In low-income countries, vaccination campaigns are lagging, and evidence on vaccine acceptance, a crucial public health planning input, remains scant. This is the first study that reports willingness to take COVID-19 vaccines and its socio-demographic correlates in Ethiopia, Africa's second most populous country. Methods: The analysis is based on a nationally representative survey data of 2,317 households conducted in the informal economy in November 2020. It employs two logistic regression models where the two outcome variables are (i) a household head's willingness to take a COVID-19 vaccine or not, and (ii) if yes if they would also hypothetically pay (an unspecified amount) for it or not. Predictors include age, gender, education, marital status, income category, health insurance coverage, sickness due to COVID-19, chronic illness, trust in government, prior participation in voluntary activities, urban residence. Results: Willingness to take the vaccine was high (88%) and significantly associated with COVID-19 cases in the family, trust in government and pro-social behavior. All other predictors such as gender, education, income, health insurance, chronic illness, urban residence did not significantly predict vaccine willingness at the 5% level. Among those willing to take the vaccine, 33% also answered that they would hypothetically pay (an unspecified amount) for it, an answer that is significantly associated with trust in government, health insurance coverage and income. Conclusion: The results highlight both opportunities and challenges. There is little evidence of vaccine hesitancy in Ethiopia among household heads operating in the informal economy. The role played by trust in government and pro-social behavior in motivating this outcome suggests that policy makers need to consider these factors in the planning of COVID-19 vaccine campaigns in order to foster vaccine uptake. At the same time, as the willingness to hypothetically pay for a COVID-19 vaccine seems to be small, fairly-priced vaccines along with financial support are also needed to ensure further uptake of COVID-19 vaccines. [ABSTRACT FROM AUTHOR]

Year	Author(s)	Title	Journal	Abstract
2021	D. R. Terefa, A. T. Shama, B. R. Feyisa, A. E. Desisa, E. T. Geta, M. C. Cheme and A. T. Edosa	COVID-19 Vaccine Uptake and Associated Factors Among Health Professionals in Ethiopia	Infection and Drug Resistance	Background: Ethiopia has received 2.2 million doses of COVID-19 vaccine from the COVID-19 Vaccines Global Access (COVAX) facility and planned to vaccinate 20% of its population by the end of 2021. However, evidence on the current uptake of the vaccine in our country is scanty. Therefore, this study aimed to assess COVID-19 vaccine uptake and associated factors among health professionals in Ethiopia. Methods: A national online cross-sectional E-survey was conducted on COVID-19 vaccine Uptake and associated factors among health professionals in Ethiopia from June 1 to 30, 2021. A semi-structured questionnaire was created on Google forms and disseminated online. The snowball sampling technique through the authors' network with Ethiopian residents on the popular social media like Facebook, telegram, and email was used. Descriptive statistics were performed. Multivariable logistic regression analysis was performed using Statistical Package for Social Sciences version 25, and all variables with P-value <0.05 and adjusted odds ratio at 95% CI were used to declare the predictors of the outcome variable. Results: A total of 522 health professionals participated in the survey, of which about 324 (62.1%) of them were vaccinated with any of the COVID-19 vaccines at least once. The study indicated that COVID-19 vaccine uptake was associated with age range from 35 to 44 years [AOR = 12.97, 95% CI: 2.36–71.21], age beyond 45 years [AOR = 18.95, 95% CI = 2.04–36.29], being male [AOR = 2.91, 95% CI = 1.05,8.09], being only an academician [AOR = 0.23, 95% CI: 0.10–0.49], academicians working in University hospitals [AOR = 0.19, 95% CI: 0.05–0.83], perceiving their family as healthy [AOR = 4.40, 95% CI: 2.21– 8.75], no history of receiving other vaccine before as an adult [AOR = 4.07, 95% CI: 2.07– 8.01] and no history of contact with confirmed COVID-19 patients or clients [AOR = 0.42, 95% CI: 0.20–0.86]. Conclusion: The study found that COVID-19 vaccine uptake among health professionals was low. This was not sufficient to achieve herd immunity as at least nine out of ten health professionals are required for herd immunity. Ages, sex, place of work, perceived family health status, previous experience of receiving a vaccine as an adult and history of contact with COVID-19 clients or patients were the factors that influence the vaccine uptake among health professionals in Ethiopia. Hence, decision makers and health managers should consider instituting mandatory vaccination for health professionals and design strategies for the provision of the vaccine. © 2021 Rikitu Terefa et al.
2022	D. Yilma, R. Mohammed, S. Getahun Abdela, W. Enbiale, F. Seifu, M. Pareyn, L. Liesenborghs, J. van Griensven and S. van Henten	COVID-19 vaccine acceptability among health care workers in Ethiopia: Do we practice what we preach?	Trop Med Int Health	OBJECTIVE: We assessed health care workers (HCWs) COVID-19 vaccine acceptability in Ethiopia. METHODS: We carried out a cross sectional survey from February to April 2021 in HCWs from five teaching hospitals. HCWs were selected using convenient sampling and data were collected through a survey link. Descriptive analysis and mixed-effect logistic regression was performed. A total of 1,314 HCWs participated in the study. RESULTS: We found that 25.5% (n=332) of the HCWs would not accept a COVID-19 vaccine and 20.2% (n=264) were not willing to recommend COVID-19 vaccination to others. Factors associated with vaccine non-acceptance were female sex (AOR=1.8; 95% CI: 1.3-2.5), the perception that vaccines are unsafe (AOR=15.0; 95% CI: 8.7-25.9), not considering COVID-19 as health risk (AOR=4.4; 95% CI: 2.0-9.5) and being unconcerned about contracting COVID-19 at work (AOR=3.5; 95% CI: 1.5-8.4). Physicians were more willing to accept vaccination than other HCWs. Higher vaccine acceptability was also noted with increasing age. Participants most often indicated safety concerns as the determining factor on their decision to get vaccinated or not. CONCLUSION: Overall, a quarter of HCWs would not accept a COVID-19 vaccine. Communications and trainings should address vaccine safety concerns. Additionally, emphasis should be given to showing current and future impact of COVID-19 on the personal, public and country level unless control efforts are improved. Interventions aimed to increase vaccine uptake should focus their efforts on younger and non-physician HCWs.
2021	Ekaete Alice Tobin, Martha Okonofua, Azuka Adeke and Andrew Obi	Willingness to accept a COVID-19 vaccine in Nigeria: a population-based cross-sectional study	Cent Afr J Public Health	

Year	Author(s)	Title	Journal	Abstract
2021	Elizabeth O. Oduwole, Tonya M. Esterhuizen, Hassan Mahomed and Charles S. Wiysonge	Estimating Vaccine Confidence Levels among Healthcare Staff and Students of a Tertiary Institution in South Africa	Vaccines	Healthcare workers were the first group scheduled to receive COVID-19 vaccines when they became available in South Africa. Therefore, estimating vaccine confidence levels and intention to receive COVID-19 vaccines among healthcare workers ahead of the national vaccination roll-out was imperative. We conducted an online survey from 4 February to 7 March 2021, to assess vaccine sentiments and COVID-19 vaccine intentions among healthcare staff and students at a tertiary institution in South Africa. We enrolled 1015 participants (74.7% female). Among the participants, 89.5% (confidence interval (CI) 87.2-91.4) were willing to accept a COVID-19 vaccine, 95.4% (CI 93.9-96.6) agreed that vaccines are important for them, 95.4% (CI 93.8-96.6) that vaccines are safe, 97.4% (CI 96.2-98.3) that vaccines are effective, and 96.1% (CI 94.6-97.2) that vaccines are compatible with religion. Log binomial regression revealed statistically significant positive associations between COVID-19 vaccine acceptance and the belief that vaccines are safe (relative risk (RR) 32.2, CI 4.67-221.89), effective (RR 21.4, CI 3.16-145.82), important for children (RR 3.5, CI 1.78-6.99), important for self (RR 18.5, CI 4.78-71.12), or compatible with religion (RR 2.2, CI 1.46-3.78). The vaccine confidence levels of the study respondents were highly positive. Nevertheless, this could be further enhanced by targeted interventions.
2021	F. A. Gbeasor-Komlanvi, K. A. Afanvi, Y. R. Konu, Y. Agbobli, A. J. Sadio, M. K. Tchankoni, W. I. C. Zida-Compaore, J. Nayo-Apetsianyi, S. Agoro, A. Lambokale, D. Nyametso, T. N'Tapi, K. Aflagah, M. Mijiyawa and D. K. Ekouevi	Prevalence and factors associated with COVID-19 vaccine hesitancy in health professionals in Togo, 2021	Public health in practice (Oxford, England)	Objectives: The aim of this study was to assess the prevalence and factors associated with COVID-19 vaccine hesitancy among health professionals (HPs) in Togo .; Study Design: Cross-sectional study.; Methods: The study was conducted between 24 February and 3 March 2021 among HPs in Togo. Data on sociodemographic characteristics and intention of vaccination were collected using an online questionnaire. Willingness to get vaccinated against COVID-19 was assessed using a single item: "Would you be willing to be vaccinated against COVID-19?". Responses were grouped into three categories: acceptance (Yes, I will get vaccinated), hesitancy (Not decided yet) and refusal (No). Multinomial regression analyses were performed to assess factors associated with vaccine hesitancy or refusal.; Results: A total of 1115 HPs (79.1% male) with a median age of 35 years were enrolled in the study. Vaccine acceptance, hesitancy and refusal were 44.1%, 32.2% and 23.7%, respectively. Female gender was associated with an increased risk of hesitancy (adjusted odds ratio [aOR] = 1.93; p = 0.005) and refusal (aOR = 1.77; p = 0.005). Participant age≥50 years, having a personal history of COVID-19 infection and a good knowledge of COVID-19 vaccination were factors that reduced the risk of refusal [(aOR = 0.30; p < 0.001), (aOR = 0.43; p = 0.031) and (aOR = 0.62; p = 0.020)] or hesitancy [(aOR = 0.53; p = 0.005), (aOR = 0.13; p < 0.001) and (aOR = 0.35; p < 0.001)] of the vaccine.; Conclusions: Acceptance of the COVID-19 vaccine before the vaccination campaign was mixed among HPs, especially young HPs. Sensitisation and information campaigns should be reinforced to combat misinformation and increase COVID-19 vaccination acceptance in the context of the ongoing global pandemic. (© 2021 The Authors.)

Year	Author(s)	Title	Journal	Abstract
2021	F. Shiferie, O. Sada, T. Fenta, M. Kaba and A. M. Fentie	Exploring reasons for COVID-19 vaccine hesitancy among healthcare providers in Ethiopia	Pan Afr Med J	INTRODUCTION: the World Health Organization has identified vaccine hesitancy as one of the top ten threats to global health. The purpose of this study was to explore factors contributing to COVID-19 vaccine hesitancy among healthcare providers, their perspectives regarding vaccine uptake by the public and their recommendations to improve vaccine uptake in Ethiopia. METHODS: a phenomenological qualitative study was conducted among purposively selected healthcare providers working in the Ministry of Health (MoH), regulatory authority, public and private hospitals and health centres who hesitated to take the COVID-19 vaccine in Addis Ababa, Ethiopia in June 2021. A total of twenty in-depth interviews were conducted using a semi-structured open-ended interview guide. Participants included nurses, physicians, pharmacists, health officers, Medical Laboratory technologists and midwives. A qualitative content analysis approach was chosen to analyse the data. RESULTS: all the participants agreed (n=20) that lack of consistent information and inadequate evidence about COVID-19 vaccine safety, efficacy and quality were the main reasons for COVID-19 vaccine hesitancy. History of perceived and confirmed COVID-19 infection history, misinformation, religious views, unknown short and long-term effects of the vaccine and undefined length of time of vaccine´s protection were also other reasons mentioned by the participants. CONCLUSION: healthcare providers were hesitant toward COVID-19 vaccine mainly due to lack of clear evidence regarding the vaccine´s short and long-term safety, efficacy and quality profiles. Hence, the long-term safety and efficacy of the vaccine should be extensively studied and evidence dissemination and communication should be clear and transparent.
2021	Felix Bongomin, Ronald Olum, Irene Andia-Biraro, Frederick Nelson Nakwagala, Khalid Hudow Hassan, Dianah Rhoda Nassozi, Mark Kaddumukasa, Pauline Byakika-Kibwika, Sarah Kiguli and Bruce J. Kirenga	COVID-19 vaccine acceptance among high-risk populations in Uganda	Therapeutic advances in infectious disease	Background: Immunization is an important strategy for controlling the COVID-19 pandemic. COVID-19 vaccination was recently launched in Uganda, with prioritization to healthcare workers and high-risk individuals. In this study, we aimed to determine the acceptability of COVID-19 vaccine among persons at high risk of COVID-19 morbidity and mortality in Uganda.; Methods: Between 29 March and 14 April 2021, we conducted a cross-sectional survey consecutively recruiting persons at high risk of severe COVID-19 (diabetes mellitus, HIV and cardiovascular disease) attending Kiruddu National Referral Hospital outpatient clinics. A trained research nurse administered a semi-structured questionnaire assessing demographics, COVID-19 vaccine related attitudes and acceptability. Descriptive statistics, bivariate and multivariable analyses were performed using STATA 16.; Results: A total of 317 participants with a mean age 51.5 ± 14.1 years were recruited. Of this, 184 (60.5%) were female. Overall, 216 (70.1%) participants were willing to accept the COVID-19 vaccine. The odds of willingness to accept COVID-19 vaccination were four times greater if a participant was male compared with if a participant was female [adjusted odds ratio (AOR): 4.1, 95% confidence interval (CI): 1.8-9.4, p = 0.00]. Participants who agreed (AOR: 0.04, 95% CI: 0.01-0.38, p = 0.003) or strongly agreed (AOR: 0.04, 95% CI: 0.01-0.59, p = 0.005) that they have some immunity against COVID-19 were also significantly less likely to accept the vaccine. Participants who had a history of vaccination hesitancy for their children were also significantly less likely to accept the COVID-19 vaccine (AOR: 0.1, 95% CI: 0.01-0.58, p = 0.016).; Conclusion: The willingness to receive a COVID-19 vaccine in this group of high-risk individuals was comparable to the global COVID-19 vaccine acceptance rate. Increased sensitization, myth busting and utilization of opinion leaders to encourage vaccine acceptability is recommended. (© The Author(s), 2021.)

Year	Author(s)	Title	Journal	Abstract
2021	G. Asmare, K. Abebe, N. Atnafu, G. Asnake, A. Yeshambel, E. Alem, E. Chekol and T. Asmamaw	Behavioral intention and its predictors toward COVID-19 vaccination among people most at risk of exposure in Ethiopia: applying the theory of planned behavior model	Hum Vaccin Immunother	Acceptance of a vaccine or hesitancy has great public health implications as these partly determine the extent to which people are exposed to infections that could have otherwise been prevented. There is a high need for a more updated understanding of the behavioral intention of the public toward COVID-19 vaccines and associated factors in light of the COVID-19 pandemic to give appropriate public health messages or actions. Thus, the study aimed to assess behavioral intention and its predictors toward COVID-19 vaccine among people most at risk of exposure in Ethiopia. A population-based anonymous online survey was conducted on individuals aged greater than 18 years from May 01, 2021 to June 30, 2021. The data were collected using a convenient sampling method through an online self-administered, structured questionnaire that was created onto Google survey tool (Google Forms) and disseminated to the public on different social media channels through online sharable platforms. Descriptive statistics were done. Bivariateand multivariable logistic regression was done to show the association of behavioral intention toward the COVID-19 vaccine. The associations of variables were declared with the use of 95% CI and P-value. A total of 1080 participants were included in this survey. Seven hundred one (64.9%) of the respondents had a behavioral intention to receive the COVID-19 vaccine. Males (AOR = 1.41 (95% CI = 1.004-2.53)), degree in level of education (AOR = 0.815 (95% CI = 0.254-0.916)), good knowledge (AOR = 4.21 (95% CI = 2.871-6.992)), attitude (AOR = 2.78 (95% CI = 1.654-4.102)), subjective norm (AOR = 1.214 (95% CI = 1.008-4.309)) and perceived behavioral control (AOR = 3.531 (95%CI = 1.689-5.201)) were found to be significantly associated with behavioral intention toward COVID-19 vaccine. Generally, the prevalence of behavioral intention in Ethiopia is low. Males, degree level of education, knowledge about vaccine, attitude toward vaccine subjective norm and perceived behavioral control were found to be significantly associated with intention to receive COVID-19 vaccine. Health education and communication from government sources are very crucial methods to alleviate the negative attitude, poor knowledge, and action need to improve or change the attitude and behavior of influential people within the community or organization to improve intention to take the vaccine.
2021	G. Murewanhema, T. V. Burukai, B. Chireka and E. Kunonga	Implementing national COVID-19 vaccination programmes in sub-Saharan Africa- early lessons from Zimbabwe: a descriptive cross-sectional study	Pan Afr Med J	INTRODUCTION: Zimbabwe was one of the first countries to run a national COVID-19 vaccination programme in Africa. Lessons learnt could inform the roll-out of similar programmes in sub-Saharan Africa. To describe the trends of uptake of the COVID-19 vaccines in the first three months (February - May 2021) of the Zimbabwe vaccination programme and the lessons learnt. METHODS: a secondary descriptive analysis of routinely available COVID-19 vaccination data extracted from the daily situation reports published by the Ministry of Health and Child Care. RESULTS: in the first three months of the programme, 1 020 078 doses were administered, with 675 678 being first doses and 344 400 were second doses. Using population estimates, at three months, 5.2% of the population had received at least one dose and 2.6% had received the full two doses. Uptake was initially slow, followed by a gradual, and subsequently an exponential increase. CONCLUSION: by the end of May 2021, Zimbabwe had rolled out one of the largest COVID-19 vaccination programme in sub-Saharan Africa. The uptake followed a pattern and trend that is consistent with vaccine hesitancy reported in the literature, driven by a combination of confidence, complacency and convenience factors. The gradual increase in uptake followed a series of national and local community engagement programmes. The roll-out of similar programmes must recognise likely patterns of uptake across the population and ensure plans are in place to address vaccine hesitancy. The available data did not allow granular analysis to understand the demographics of people who participated in the programme, which is important for surveillance, targeted action, preventing inequalities and ensuring adequate and proportionate protection of residents prioritising the most vulnerable. Further analysis of the process, outcomes and impact of the programme will be helpful in informing the roll-out of similar programmes across Africa.

Year	Author(s)	Title	Journal	Abstract
2022	H. Adedeji-Adenola, O. A. Olugbake and S. A. Adeosun	Factors influencing COVID-19 vaccine uptake among adults in Nigeria	PLoS One	BACKGROUND: Emerging variants of Coronavirus disease 2019 (COVID-19) has claimed over 3000 lives in Nigeria and vaccination remains a means of reducing the death toll. Despite ongoing efforts by the government to ensure COVID-19 vaccination of most residents to attain herd immunity, myths and beliefs have adversely shaped the perception of most Nigerians, challenging the uptake of COVID-19 vaccine. This study aimed to assess the factors influencing the awareness, perception, and willingness to receive COVID-19 vaccine among Nigerian adults. METHODS: A cross-sectional online nationwide study was conducted from April to June 2021 among Nigerian adult population using the snowballing method. Descriptive analysis was used to summarise the data. Univariate and multivariate analysis was used to identify the predictors of COVID-19 uptake among the respondents. A p value <0.05 was considered significant. RESULTS: A total of 1058 completed forms were analysed and 63.9% were females. The mean age was 40.8 years±12.2 years. Most of the respondents (740; 69.5%) had satisfactory awareness of the vaccination exercise. The media was the main source of information. Health workers reported higher level of awareness (aOR = 1.822, 95% CI: 1.388-2.524, p<0.001). Respondents that are Christians and Muslims had better awareness compared to the unaffiliated (aOR = 6.398, 95% CI: 1.918-21.338, P = 0.003) and (aOR = 7.595, 95% CI: 2.280-25.301, p<0.001) respectively. There is average score for perception statements (566; 53.2%) towards COVID-19 vaccination. Close to half of the respondents (44.2%) found the short period of COVID-19 production worrisome. Majority of the respondents were willing to get the vaccine (856; 80.9%). Those without a prior diagnosis of COVID-19 had a lower willingness to get vaccinated (aOR = 0.210 (95% CI: 0.082-0.536) P = 0.001). CONCLUSION: The study revealed a high level of awareness, willingness to receive the vaccine and moderate perception towards the vaccination activities. Influencing factors that significantly affects awareness were religion, occupation, education and prior diagnosis of COVID-19; for perception and willingness-occupation, and prior diagnosis of the COVID-19 were influencing factors.
2021	Haimanot Abebe, Solomon Shitu and Ayenew Mose	Understanding of COVID-19 vaccine knowledge, attitude, acceptance, and determinates of COVID-19 vaccine acceptance among adult population in Ethiopia	Infection and drug resistance	
2021	J. D. Ditekemena, D. M. Nkamba, A. Mutwadi, H. M. Mavoko, J. N. Siewe Fodjo, C. Luhata, M. Obimpeh, S. Van Hees, J. B. Nachega and R. Colebunders	COVID-19 Vaccine Acceptance in the Democratic Republic of Congo: A Cross-Sectional Survey	Vaccines (Basel)	We investigated the level of willingness for COVID-19 vaccination in the Democratic Republic of Congo (DRC). Data were collected between 24 August 2020 and 8 September 2020 through an online survey. A total of 4131 responses were included; mean age of respondents was 35 years (standard deviation: 11.5); 68.4% were females; 71% had elementary or secondary school education. One fourth (24.1%) were convinced that COVID-19 did not exist. Overall, 2310 (55.9%) indicated they were willing to be vaccinated. In a multivariable regression model, belonging to the middle and high-income category (OR = 1.85, CI: 1.46-2.35 and OR = 2.91, CI: 2.15-3.93, respectively), being tested for COVID-19 (OR = 4.71, CI: 3.62-6.12; p < 0.001), COVID-19 community vaccine acceptance (OR = 14.45, CI: 2.91-71.65; p = 0.001) and acknowledging the existence of COVID-19 (OR = 6.04, CI: 4.42-8.23; p < 0.001) were associated with an increased willingness to be vaccinated. Being a healthcare worker was associated with a decreased willingness for vaccination (OR = 0.46, CI: 0.36-0.58; p < 0.001). In conclusion, the current willingness for COVID-19 vaccination among citizens of the DRC is too low to dramatically decrease community transmission. Of great concern is the low intention of immunization among healthcare workers. A large sensitization campaign will be needed to increase COVID-19 vaccine acceptance.

Year	Author(s)	Title	Journal	Abstract
2021	J. Dula, A. Mulhanga, A. Nhanombe, L. Cumbi, A. Júnior, J. Gwatsvaira, J. N. Siewe Fodjo, E. F. De Moura Villela, S. Chicumbe and R. Colebunders	Covid-19 vaccine acceptability and its determinants in mozambique: An online survey	Vaccines	A high worldwide SARS-CoV-2 vaccine coverage must be attained to stop the COVID-19 pandemic. In this study, we assessed the level of willingness of Mozambicans to be vaccinated against COVID-19. Data were collected between 11 and 20 March 2021, through a self-administered online survey. Of the 1878 respondents, 30.1% were healthcare workers, 58.3% were aged between 18 and 35 years, 60% were male, and 38.5% were single. Up to 43% had been tested for COVID-19 and 29% had tested positive. Overall vaccine acceptability was 71.4% (86.6% among healthcare workers, 64.8% among other respondents, p < 0.001). Reasons for vaccine hesitancy included: Fear of vaccine side effects (29.6%) and the belief that the vaccine is not effective (52%). The acceptability of the SARSCoV-2 vaccine increased with increasing vaccine efficacy. Using logistic regression, determinants for acceptability of the vaccine were: Older age, a past COVID-19 test, a concern of becoming (re)infected by COVID-19, having a chronic disease, and considering vaccination important for personal and community health. In conclusion, vaccine acceptability in Mozambique was relatively high among healthcare workers but significantly lower in the rest of the population. This suggests that there is a need to educate the general population about SARS-CoV-2 vaccination and its importance. © 2021 by the authors.
2020	Jeffrey V. Lazarus, Katarzyna Wyka, Lauren Rauh, Kenneth Rabin, Scott Ratzan, Lawrence O. Gostin, Heidi J. Larson and Ayman El-Mohandes	Hesitant or not? The association of age, gender, and education with potential acceptance of a COVID-19 vaccine: A country-level analysis	Journal of Health Communication	In December 2020, the first COVID-19 vaccines were approved. Despite more than 85 million reported cases and 1.8 million known deaths, millions worldwide say they may not accept it. This study assesses the associations of age, gender, and level of education with vaccine acceptance, from a random sample of 13,426 participants selected from 19 high-COVID-19 burden countries in June 2020. Based on univariable and multivariable logistic regression, several noteworthy trends emerged: women in France, Germany, Russia, and Sweden were significantly more likely to accept a vaccine than men in these countries. Older (≥50) people in Canada, Poland, France, Germany, Sweden, and the UK were significantly more favorably disposed to vaccination than younger respondents, but the reverse trend held in China. Highly educated individuals in Ecuador, France, Germany, India, and the US reported that they will accept a vaccine, but higher education levels were associated with lower vaccination acceptance in Canada, Spain, and the UK. Heterogeneity by demographic factors in the respondents' willingness to accept a vaccine if recommended by employers were substantial when comparing responses from Brazil, Ecuador, France, India, Italy, Mexico, Poland, Russia, South Africa, South Korea, Sweden, and the US. This information should help public health authorities target vaccine promotion messages more effectively. (PsycInfo Database Record (c) 2021 APA, all rights reserved)

Year	Author(s)	Title	Journal	Abstract
2021	Jerome Nyhalah Dinga, Leontine Kouemou Sinda and Vincent P. K. Titanji	Assessment of Vaccine Hesitancy to a COVID-19 Vaccine in Cameroonian Adults and Its Global Implication	Vaccines	Since the outbreak of COVID-19 in December 2019, no global consensus treatment has been developed and generally accepted for the disease. However, eradicating the disease will require a safe and efficacious vaccine. In order to prepare for the eventual development of a safe and efficacious COVID-19 vaccine and to enhance its uptake, it is imperative to assess vaccine hesitancy in Cameroonians. After obtaining ethical clearance from the Institutional Review Board of the University of Buea, a questionnaire was administered (May-August 2020) to consenting adults either online or in person. A qualitative thematic analysis was done to analyze the participants' answers to the open questions. A deductive approach was used, that is, the codes and patterns according to the World Health Organization (WHO) Strategic Advisory Group of Experts (SAGE) Working Group Matrix of Determinants of vaccine hesitancy. The number of consenting adult Cameroonians who completed the questionnaire were 2512 (Two thousand five hundred and twelve). Vaccine hesitancy to a COVID-19 vaccine was 84.6% in Cameroonians. Using the WHO recommended Matrix of Determinant of Vaccine hesitancy, the most prominent determinants observed in this study were: Communication and Media Environment, Perception of pharmaceutical industry, Reliability and/or source of vaccine and cost. Most Cameroonians agree that even though there are benefits of a clinical trial, they will prefer it should be done out of the continent and involving African scientists for eventual acceptance and uptake. The concerns of safety, efficacy and confidence has to be addressed using a Public Engagement approach if a COVID-19 vaccine has to be administered successfully in Africa or Cameroon specifically. Since this study was carried out following WHO standards, its result can be compared to those of other studies carried out in different cultural settings using similar standards.
2021	Julio S Solís Arce, Shana S Warren, Niccolò F Meriggi, Alexandra Scacco, Nina McMurry, Maarten Voors, Georgiy Syunyaev, Amyn Abdul Malik, Samya Aboutajdine and Opeyemi Adeojo	COVID-19 vaccine acceptance and hesitancy in low- and middle-income countries	Nature medicine	
2021	Kegnie Shitu, Maereg Wolde, Simegnew Handebo and Ayenew Kassie	Acceptance and willingness to pay for COVID-19 vaccine among school teachers in Gondar City, Northwest Ethiopia	Tropical medicine and health	

Year	Author(s)	Title	Journal	Abstract
2021	Lauren McAbee, Oscar Tapera and Mufaro Kanyangarara	Factors Associated with COVID-19 Vaccine Intentions in Eastern Zimbabwe: A Cross-Sectional Study	Vaccines	Vaccines are one of the most effective public health strategies to protect against infectious diseases, yet vaccine hesitancy has emerged as a global health threat. Understanding COVID-19 knowledge and attitudes and their association with vaccine intentions can help the targeting of strategies to increase vaccination uptake and achieve herd immunity. The goal of this study was to assess COVID-19 knowledge, attitudes, and behaviors, and identify factors associated with COVID-19 vaccine intentions among heads of households in Manicaland Province, Zimbabwe. A cross-sectional survey was conducted in May 2021 among 551 randomly selected households. Data were collected on socio-demographic characteristics, and knowledge, attitudes, and behaviors regarding COVID-19 and the vaccines. More than half (55.7%) of the respondents reported intending to vaccinate themselves or their households. Multivariate logistic regression indicated that the likelihood of vaccine intentions was most strongly associated with confidence in vaccine safety. Additionally, the odds of intending to get vaccinated were significantly higher among heads of households who were male, had a higher level of education, and identified vaccination and face mask usage as prevention measures. Among perceived motivators to vaccinate, recommendations from the World Health Organization and availability of the vaccine free of charge increased the likelihood of vaccine intentions, while country of vaccine manufacturer posed a barrier to vaccine intentions. As the vaccine rollout in Zimbabwe continues, efforts to increase COVID-19 vaccination coverage and achieve herd immunity should target females and less educated populations and be tailored to address concerns about vaccine safety and country of manufacturer.
2022	Lulin Zhou, Sabina Ampon-Wireko, Xinglong Xu, Prince Edwudzie Quansah and Ebenezer Larnyo	Media attention and Vaccine Hesitancy: Examining the mediating effects of Fear of COVID-19 and the moderating role of Trust in leadership	PLoS ONE	Vaccination has emerged as the most cost-effective public health strategy for maintaining population health, with various social and economic benefits. These vaccines, however, cannot be effective without widespread acceptance. The present study examines the effect of media attention on COVID-19 vaccine hesitancy by incorporating fear of COVID-19 as a mediator, whereas trust in leadership served as a moderator. An analytical cross-sectional study is performed among rural folks in the Wassa Amenfi Central of Ghana. Using a questionnaire survey, we were able to collect 3079 valid responses. The Smart PLS was used to estimate the relationship among the variables. The results revealed that media attention had a significant influence on vaccine hesitancy. Furthermore, the results showed that fear of COVID-19 played a significant mediating role in the relationship between media and vaccine hesitancy. However, trust in leadership had an insignificant moderating relationship on the fear of COVID-19 and vaccine hesitancy. The study suggests that the health management team can reduce vaccine hesitancy if they focus on lessening the negative impact of media and other antecedents like fear on trust in leadership. [ABSTRACT FROM AUTHOR]

Year	Author(s)	Title	Journal	Abstract
2021	M. A. M. Ahmed, R. Colebunders, A. A. Gele, A. A. Farah, S. Osman, I. A. Guled, A. A. M. Abdullahi, A. M. Hussein, A. M. Ali and J. N. S. Fodjo	COVID-19 Vaccine Acceptability and Adherence to Preventive Measures in Somalia: Results of an Online Survey	Vaccines	Most countries are currently gravitating towards vaccination as mainstay strategy to quell COVID-19 transmission. Between December 2020 and January 2021, we conducted a follow-up online survey in Somalia to monitor adherence to COVID-19 preventive measures, and COVID-19 vaccine acceptability and reasons for vaccine hesitancy. Adherence was measured via a composite adherence score based on four measures (physical distancing, face mask use, hand hygiene, and mouth covering when coughing/sneezing). We analyzed 4543 responses (mean age: 23.5 ± 6.4 years, 62.4% males). The mean adherence score during this survey was lower than the score during a similar survey in April 2020. A total of 76.8% of respondents were willing to receive the COVID-19 vaccine. Flu-like symptoms were more frequently reported in the current survey compared to previous surveys. Multiple logistic regression showed that participants who experienced flu-like symptoms, those in the healthcare sector, and those with higher adherence scores had higher odds for vaccine acceptability while being a female reduced the willingness to be vaccinated. In conclusion, our data suggest that the decreasing adherence to COVID-19 preventive measures may have caused increased flu-like symptoms over time. COVID-19 vaccine acceptance in Somalia is relatively high but could be improved by addressing factors that contribute to vaccine hesitancy.
2021	M. G. Salomoni, Z. Di Valerio, E. Gabrielli, M. Montalti, D. Tedesco, F. Guaraldi and D. Gori	Hesitant or not hesitant? A systematic review on global covid-19 vaccine acceptance in different populations	Vaccines	Vaccination currently appears to be the only strategy to contain the spread of COVID-19. At the same time, vaccine hesitancy (VH) could limit its efficacy and has, therefore, attracted the attention of Public Health Systems. This systematic review aimed at assessing anti-COVID-19 vaccine acceptance rates worldwide and at identifying populations more prone to vaccine hesitancy, for which specific interventions should be planned. PubMed database was searched using a purposely formulated string. One hundred out of the 9243 studies retrieved were considered pertinent and thus included in the analyses. VH rate was analyzed according to patient geographical origin, ethnicity, age, study setting, and method used for data collection; data from specific populations were separately analyzed. Overall, this study demonstrated significant differences in terms of VH in the general population and in the specific subgroups examined according to geographical, demographic factors, as well as associated comorbidities, underlining the need for purposely designed studies in specific populations from the different countries, to design targeted programs aimed at increasing awareness for confidence and complacency toward COVID-19 vaccines. © 2021 by the authors. Licensee MDPI, Basel, Switzerland.
2020	M. Kabamba Nzaji, L. Kabamba Ngombe, G. Ngoie Mwamba, D. B. Banza Ndala, J. Mbidi Miema, C. Luhata Lungoyo, B. Lora Mwimba, A. Cikomola Mwana Bene and E. Mukamba Musenga	Acceptability of Vaccination Against COVID-19 Among Healthcare Workers in the Democratic Republic of the Congo	Pragmat Obs Res	PURPOSE: This study aims to estimate the acceptability of a future vaccine against COVID-19 and associated factors if offered in Congolese health-care workers (HCWs), since they have the highest direct exposure to the disease. PATIENTS AND METHODS: We conducted an analytical cross-sectional study among 23 Congolese referral hospitals, including three university hospitals, located in three towns from March through 30 April 2020. The main outcome variable was healthcare workers' acceptance of a future vaccine against COVID-19. The associated factors of vaccination willingness were identified through a logistic regression analysis. RESULTS: A sample of 613 HCWs participated in the study and completed the study questionnaire, including 312 (50.9%) men and 301 (49.1%) women. Only 27.7% of HCWs said that they would accept a COVID-19 vaccine if it was available. From the logistic regression analysis, male healthcare workers (ORa=1.17, 95% CI: 1.15-2.60), primarily doctors (ORa=1.59; 95% CI:1.03-2.44) and having a positive attitude towards a COVID-19 vaccine (ORa=11.49; 95% CI: 5.88-22.46) were significantly associated with reporting willingness to be vaccinated. CONCLUSION: For acceptability of vaccination against COVID-19 among others education among HCWs is crucial because health professionals' attitudes about vaccines are an important determinant of their own vaccine uptake and their likelihood of recommending the vaccine to their patients.

Year	Author(s)	Title	Journal	Abstract
2021	M. Mesesle	Awareness and attitude towards covid-19 vaccination and associated factors in ethiopia: Cross-sectional study	Infection and Drug Resistance	Background: Coronavirus disease (COVID-19) is a deadly virus that continues to afflict many countries worldwide. The development of a COVID-19 vaccine to combat the disease's spread and devastating effects is still ongoing, and as the pandemic progresses, new, more effective vaccines are likely to be created. The aim of this study was to assess awareness and attitude towards COVID-19 vaccination in Ethiopia. Methods: A population-based cross-sectional e-survey was conducted among 425 participants from March 13, 2021 to April 10, 2021. The survey was conducted using a structured and self-reported questionnaire containing informed consent along with three sections (sociodemographic, awareness, and attitude); a multivariable logistic regression model was performed to determine the variables predicting awareness towards COVID-19 vaccinations. Results: The mean score of awareness was 4.3 (SD=1.1) out of 7, with the overall awareness of 40.8%, and the mean score of attitudes was 4.09 (SD=2.16) out of 9, with an overall "positive attitude" score of 24.2%. College and above educational level (AOR=2.21, 95% CI=1.32, 4.62), had access to mass media (AOR=4.75, 95% CI =2.74, 8.24), and urban residency (AOR=2.83, 95% C.I = 1.57, 5.09) were significantly associated with awareness towards COVID-19 vaccination. Conclusion: In Ethiopia, there is a poor knowledge toward COVID-19 vaccines, according to the current report. The findings indicate that authorities should implement an urgent health education program and disseminate more reliable information. Using the media, policymakers should take measures to ensure adequate awareness of COVID-19 vaccinations with various stakeholders. © 2021 Mesesle.
2021	M. T. J. Ansari and N. A. Khan	Worldwide COVID-19 vaccines sentiment analysis through twitter content	Electronic Journal of General Medicine	One year during the pandemic of COVID 19, numerous viable possibilities have been created in worldwide efforts to create and disseminate a viable vaccine. The rapid development of numerous vaccinations is remarkable; generally, the procedure takes 8 to 15 years. The vaccination of a critical proportion of the global population, which is vital for containing the pandemic, is now facing a new set of hurdles, including hazardous new strains of the virus, worldwide competition over a shortage of doses, as well as public suspicion about the vaccinations. A safe and efficacious vaccine COVID-19 is borne fruit globally. There are presently more than a dozen vaccinations worldwide authorized; many more continue to be developed. This paper used COVID-19 vaccine related tweets to present an overview of the public's reactions on current vaccination drives by using thematic sentiment and emotional analysis, and demographics interpretation to people. Further, experiments were carried out for sentiment analysis in order to uncover fresh information about the effect of location and gender. Overall Tweets were generally negative in tone and a huge vaccination trend can be seen in global health perspectives, as evidenced by the analysis of the role of comprehensive science and research in vaccination. © 2021 by Author/s and Licensed by Modestum.
2021	Malik Sallam	COVID-19 Vaccine Hesitancy Worldwide: A Concise Systematic Review of Vaccine Acceptance Rates	Vaccines	
2021	Martin Wiredu Agyekum, Grace Frempong Afrifa-Anane, Frank Kyei-Arthur and Bright Addo	Acceptability of COVID-19 vaccination among health care workers in Ghana	Advances in Public Health	

Year	Author(s)	Title	Journal	Abstract
2022	Melissa Leach, Hayley MacGregor, Grace Akello, Lawrence Babawo, Moses Baluku, Alice Desclaux, Catherine Grant, Foday Kamara, Marion Nyakoi, Melissa Parker, Paul Richards, Esther Mokuwa, Bob Okello, Kelley Sams and Khoudia Sow	Vaccine anxieties, vaccine preparedness: Perspectives from Africa in a Covid-19 era	Social Science & Medicine	Global debates about vaccines as a key element of pandemic response and future preparedness in the era of Covid-19 currently focus on questions of supply, with attention to global injustice in vaccine distribution and African countries as rightful beneficiaries of international de-regulation and financing initiatives such as COVAX. At the same time, vaccine demand and uptake are seen to be threatened by hesitancy, often attributed to an increasingly globalised anti-vaxx movement and its propagation of misinformation and conspiracy, now reaching African populations through a social media 'infodemic'. Underplayed in these debates are the socio-political contexts through which vaccine technologies enter and are interpreted within African settings, and the crucial intersections between supply and demand. We explore these through a 'vaccine anxieties' framework attending to both desires for and worries about vaccines, as shaped by bodily, societal and wider political understandings and experiences. This provides an analytical lens to organise and interpret ethnographic and narrative accounts in local and national settings in Uganda and Sierra Leone, and their (dis)connections with global debates and geopolitics. In considering the socially-embedded reasons why people want or do not want Covid-19 vaccines, and how this intersects with the dynamics of vaccine supply, access and distribution in rapidly-unfolding epidemic situations, we bring new, expanded insights into debates about vaccine confidence and vaccine preparedness. (Copyright © 2022 The Authors. Published by Elsevier Ltd.. All rights reserved.)
2022	Metadel Adane, Ayechew Ademas and Helmut Kloos	Knowledge, attitudes, and perceptions of COVID-19 vaccine and refusal to receive COVID-19 vaccine among healthcare workers in northeastern Ethiopia	BMC Public Health	<bold>Background: </bold>Major efforts are being made to control the spread and impacts of the coronavirus pandemic using vaccines. Ethiopia began on March 13, 2021, to vaccinate healthcare workers (HCWs) for COVID-19 with the AstraZeneca vaccine. However, willingness to be vaccinated depends to a large extent on factors beyond the availability of vaccines. This study aimed to determine the rate of intention to refuse COVID-19 vaccination and associated factors among HCWs in northeastern Ethiopia. northeastern, Ethiopia.<bold>Method: </bold>An institution-based cross-sectional study was employed among 404 HCWs in Dessie City, northeastern Ethiopia in May, 2021. Data were collected, checked, coded, entered into EpiData Version 4.6 and exported to Statistical Package of Social Sciences (SPSS) Version 25.0 for cleaning and analysis. The dependent variable was refuse to receive COVID-19 vaccination and the independent variables included socio-demographic factors, knowledge, attitudes and perceptions. A Binary logistic regression model was used to determine the association between vaccine refusal and the independent variables. From bivariate analysis, variables with p-values < 0.25 were retained for multivariable analysis. From multivariable analysis, variables with adjusted odds ratio (AOR), p-values <0.05 at 95% confidence interval (CI) were declared as factors significantly associated with refusal to be vaccinated among HCWs in Dessie City, northeastern Ethiopia.<bold>Results: </bold>The proportion of HCWs with overall good knowledge, good perception, and positive attitudes about COVID-19 vaccination were 62.5%, 60.5%, and 52.3%, respectively; 64.0% of the HCWs wanted to be vaccinated while 36.0% said that they would refuse to do so. Multivariable analysis identified negative attitudes (AOR: 3.057; 95%CI [1.860 - 5.026]) and poor perceptions (AOR: 4.73; 95%CI [2.911 - 7.684]) about COVID-19 vaccines were significantly associated with refusal to be vaccinated for COVID-19. Nearly half (46.9%) of the HCWs stated that vaccines could worsen any pre-existing medical conditions and 39.5% of them thought that vaccines could cause COVID-19 infections.<bold>Conclusion: </bold>The willingness of HCWs to get vaccinated against COVID-19 was relatively high among HCWs. Negative attitudes and poor perceptions towards the anticipated COVID-19 vaccination were significant factors to refuse to be vaccinated. Our findings may provide information for the management authorities and stakeholders to promote and improve attitudes, knowledge and perceptions towards COVID-19 vaccination uptake among HCWs. [ABSTRACT FROM AUTHOR]

Year	Author(s)	Title	Journal	Abstract
2021	Mohammed A. M. Ahmed, Robert Colebunders, Abdi A. Gele, Abdiqani A. Farah, Shariff Osman, Ibraahim Abdullahi Guled, Aweis Ahmed Moalim Abdullahi, Ahmed Mohamud Hussein, Abdiaziz Mohamed Ali and Joseph Nelson Siewe Fodjo	COVID-19 Vaccine Acceptability and Adherence to Preventive Measures in Somalia: Results of an Online Survey	Vaccines	
2021	Nasim Asadi Faezi, Pourya Gholizadeh, Moussa Sanogo, Amadou Oumarou, Maad Nasser Mohamed, Yacouba Cissoko, Mamadou Saliou Sow, Bakary Sayon Keita, Youssouf A. G. Mohamed Baye, Pasquale Pagliano, Patassi Akouda, Sid'Ahmed Soufiane, Akory Ag Iknane, Mamadou Oury Safiatou Diallo, Zakaria Gansane, Barkat Ali Khan, Şükran Köse, Hamid Allahverdipour, Khudaverdi Ganvarov and Mariam Soumaré	Peoples' attitude toward COVID-19 vaccine, acceptance, and social trust among African and Middle East countries	Health Promotion Perspectives	Background: To end the COVID-19 pandemic, a large part of the world must be immune to the virus by vaccination. Therefore, this study aimed to gauge intent to be vaccinated against COVID-19 among ordinary people and to identify attitudes towards vaccines and barriers for vaccine acceptance. Methods: The study population comprises 1880 people residing in different countries that answer a prepared questionnaire. The questionnaire topics are demographics, historical issues, participants' attitudes and beliefs regarding vaccines, concerns, and vaccine hesitancy. Results: Attitudes and beliefs relating to vaccines in general, and the COVID-19 vaccine, were ascertained. Overall, 66.81% of the contributors would like to be vaccinated against COVID-19, while %33.19 did not intend to be vaccinated. Reasons for COVID-19 vaccine hesitancy included concern regarding vaccine side effects, fear of getting sick from the uptake of the vaccine, and the absence of accurate vaccine promotion news. Individuals with higher education believe that India (68.6%) produces the best vaccine (P <0.001), while healthcare workers think the Chinese vaccine (44.2%) is the best (P =0.020). Individuals with higher education have not been vaccinated, not be healthcare workers, and females were the most contributors to effective of the vaccine in reducing mortality from COVID-19 disease. Conclusion: Given the degree of hesitancy against COVID-19 vaccination, a multifaceted approach to facilitate vaccine uptake that includes vaccine education, behavioral change strategies, and health promotion, is paramount. [ABSTRACT FROM AUTHOR]
2021	O. V. Adeniyi, D. Stead, M. Singata-Madliki, J. Batting, M. Wright, E. Jelliman, S. Abrahams and A. Parrish	Acceptance of covid-19 vaccine among the healthcare workers in the eastern cape, south africa: A cross sectional study	Vaccines	Background: This study assesses the perceptions and acceptance of severe acute respiratory syndrome coronavirus-2 (SARS-CoV-2) vaccination. It also examines its influencing factors among the healthcare workers (HCWs) in the Eastern Cape, South Africa. Methods: In this cross-sectional study performed in November and December 2020, a total of 1308 HCWs from two large academic hospitals participated in the Eastern Cape Healthcare Workers Acquisition of SARS-CoV-2 (ECHAS) study. Validated measures of vaccine hesitancy were explored using a questionnaire. Logistic regression was used to identify the determinants of vaccine hesitancy. Results: The majority were nurses (45.2%), and at risk for unfavourable Covid-19 outcome, due to obesity (62.9%) and having direct contact with individuals confirmed to have Covid-19 (77.1%). The overall acceptance of SARS-CoV-2 vaccine was 90.1%, which differed significantly by level of education. Individuals with lower educational attainment (primary and secondary education) and those with prior vaccine refusal were less likely to accept the SARS-CoV-2 vaccine. However, positive perceptions about the SARS-CoV-2 vaccine were independently associated with vaccine acceptance. Conclusions: The high level of acceptance of SARS-CoV-2 vaccine is reassuring; however, HCWs with a lower level of education and those with prior vaccine refusal should be targeted for further engagements to address their concerns and fears. © 2021 by the authors. Licensee MDPI, Basel, Switzerland.

Year	Author(s)	Title	Journal	Abstract
2021	Obi Peter Adigwe	COVID-19 vaccine hesitancy and willingness to pay: Emergent factors from a cross-sectional study in Nigeria	Vaccine: X	Introduction: Prior to the COVID-19 pandemic, it took at least several years to develop vaccines for prevention of infectious diseases. The COVID-19 vaccine is the first to be developed within a period of one year. The expediency associated with the development of the COVID-19 vaccine has however been hampered by vaccine hesitancy and other relevant factors that could influence consequent immunisation. This study aimed at investigating factors associated with vaccine hesitancy and willingness to pay for COVID-19 vaccination.; Methods: A cross-sectional approach was used to undertake online and physical data collection with a validated questionnaire.; Results: A total of 1767 valid responses were received, female participants were in the minority (42.2%), majority (54.9%) of the study participants were between the ages of 18 and 30 years, and more than half (53.8%) of the participants were educated up to first degree level. Slightly above half (52.9%) of the study participants indicated that they were worried about side effects that may be associated with COVID-19 vaccines, and this may likely prevent them from taking the vaccine. A strong majority (85.1%) of the study participants indicated that COVID-19 vaccine should be administered at no cost to citizens. Only a quarter (26%) of the participants were willing to pay a fee for COVID-19 vaccination. Also, older participants and those that had been previously infected with COVID-19 were more likely to pay for COVID-19 vaccination.; Conclusion: This study provides critical insights which could influence immunisation efforts during the pandemic. An early understanding of population perceptions of the COVID-19 vaccine can be invaluable in designing successful campaigns. This is even more critical, given supply limitations, access issues and vaccines' inequity occasioned by the international scramble. (© 2021 The Author.)
2021	P. C. Addo, N. B. Kulbo, K. A. Sagoe, A. A. Ohemeng and E. Amuzu	Guarding against COVID-19 vaccine hesitance in Ghana: analytic view of personal health engagement and vaccine related attitude	Hum Vaccin Immunother	Vaccination is the most effective preventive measure against COVID-19 spread. While the WHO and other stakeholders fear vaccine nationalism, vaccine-hesitancy has become a topical issue among experts. Based on the evidence of vaccine hesitancy among Blacks, we explore the interrelatedness of psycho-social factors (personal health engagement, fear of COVID-19, perceived susceptibility, and vaccine-related attitude) likely to thwart vaccine acceptance in Africa. We sampled 1768 Ghanaian adults over 2 weeks from December 14, 2020, the first day a successful COVID-19 vaccine was administered in the US using an online survey. A higher level of personal health engagement was found to promote vaccine-related attitudes while reducing COVID-19 related fears, susceptibility, and vaccine hesitancy. Fear of COVID-19 and perceived vulnerability are significant contributors to the willingness to accept vaccination. This is an indication that health engagement alone will not promote vaccination willingness, but the fear and higher level of perceived susceptibility out of personal evaluation are essential factors in vaccination willingness. We recommend promoting health educational messages on COVID-19 vaccination ahead of any vaccination rollout in Africa, and such messages should contain some element of fear appeal.
2022	Patrick D. M. C. Katoto, Saahier Parker, Nancy Coulson, Nirvana Pillay, Sara Cooper, Anelisa Jaca, Edison Mavundza, Gregory Houston, Candice Groenewald, Zaynab Essack, Jane Simmonds, Londiwe Deborah Shandu, Marilyn Couch, Nonkululeko Khuzwayo, Nobukhosi Ncube, Phelele Bhengu, Heidi van Rooyen and Charles Shey Wiysonge	Predictors of COVID-19 Vaccine Hesitancy in South African Local Communities: The VaxScenes Study	Vaccines	

Year	Author(s)	Title	Journal	Abstract
2021	Rihanna Mohammed, Teklehaimanot Mezgebe Nguse, Bruck Messele Habte, Atalay Mulu Fentie and Gebremedhin Beedemariam Gebretekle	COVID-19 vaccine hesitancy among Ethiopian healthcare workers	PLoS ONE	Introduction: COVID-19 poses significant health and economic threat prompting international firms to rapidly develop vaccines and secure quick regulatory approval. Although COVID-19 vaccination priority is given for high-risk individuals including healthcare workers (HCWs), the success of the immunization efforts hinges on peoples' willingness to embrace these vaccines. Objective: This study aimed to assess HCWs intention to be vaccinated against COVID-19 and the reasons underlying vaccine hesitancy. Methods: A cross-sectional survey was conducted among HCWs in Addis Ababa, Ethiopia from March to July 2021. Data were collected from eligible participants from 18 health facilities using a pre-tested semi-structured questionnaire. Data were summarized using descriptive statistics and multivariable logistic regression was performed to explore factors associated with COVID-19 vaccine hesitancy. A p<0.05 was considered statistically significant. Results: A total of 614 HCWs participated in the study, with a mean age of 30.57±6.87 years. Nearly two-thirds (60.3%) of HCWs were hesitant to use the COVID-19 vaccine. Participants under the age of 30 years were approximately five times more likely to be hesitant to be vaccinated compared to those over the age of 40 years. HCWs other than medical doctors and/or nurses (AOR = 2.1; 95%CI; 1.1, 3.8) were more likely to be hesitant for COVID-19 vaccine. Lack of believe in COVID-19 vaccine benefits (AOR = 2.5; 95%CI; 1.3, 4.6), lack of trust in the government (AOR = 1.9; 95%CI; 1.3, 3.1), lack of trust science to produce safe and effective vaccines (AOR = 2.6; 95%CI; 1.6, 4.2); and concern about vaccine safety (AOR = 3.2; 95%CI; 1.9, 5.4) were also found to be predictors of COVID-19 vaccine hesitancy. Conclusion: COVID-19 vaccine hesitancy showed to be high among HCWs. All concerned bodies including the ministry, regional health authorities, health institutions, and HCWs themselves should work together to increase COVID-19 vaccine uptake and overcome the pandemic. [ABSTRACT FROM AUTHOR]
2021	Robert Kaba Alhassan, Matilda Aberese-Ako, Phidelia Theresa Doegah, Mustapha Immurana, Maxwel Ayindenaba Dalaba, Alfred Kwesi Manyeh, Desmond Klu, Evelyn Acquah, Evelyn Korkor Ansah and Margaret Gyapong	COVID-19 vaccine hesitancy among the adult population in Ghana: evidence from a pre-vaccination rollout survey	Tropical Medicine & Health	Background: Coronavirus disease 2019 (COVID-19) has already claimed over four million lives globally and over 800 deaths in Ghana. The COVID-19 vaccine is a key intervention towards containing the pandemic. Over three billion doses of the vaccine have already been administered globally and over 800,000 doses administered in Ghana, representing less than 5% vaccination coverage. Fear, uncertainty, conspiracy theories and safety concerns remain important threats to, a successful rollout of the vaccine if not managed well. Objective: Ascertain the predictors of citizens' probability of participating in a COVID-19 vaccine trial and subsequently accept the vaccine when given the opportunity. Methodology: The study was an online nation-wide survey among community members (n = 1556) from 18th September to 23rd October, 2020 in the 16 regions in Ghana. Binary probit regression analysis with marginal effect estimations was employed to ascertain the predictors of community members' willingness to participate in a COVID-19 vaccine trial and uptake the vaccine. Results: Approximately 60% of respondents said they will not participate in a COVID-19 vaccine trial; 65% will take the vaccine, while 69% will recommend it to others. Willingness to voluntarily participate in COVID-19 vaccine trial, uptake the vaccine and advise others to do same was higher among adults aged 18–48 years, the unmarried and males (p < 0.05). Significant predictors of unwillingness to participate in the COVID-19 vaccine trial and uptake of the vaccine are: married persons, females, Muslims, older persons, residents of less urbanised regions and persons with lower or no formal education (p < 0.05). Predominant reasons cited for unwillingness to participate in a COVID-19 vaccine trial and take the vaccine included fear, safety concerns, lack of trust in state institutions, uncertainty, political connotations, spiritual and religious beliefs. Conclusion: The probability of accepting COVID-19 vaccine among the adult population in Ghana is high but the country should not get complacent because fear, safety and mistrust are important concerns that have the potential to entrench vaccine hesitancy. COVID-19 vaccine rollout campaigns should be targeted and cognisant of the key predictors of citizens' perceptions of the vaccine. These lessons when considered will promote Ghana's efforts towards vaccinating at least 20 million people to attain herd immunity. [ABSTRACT FROM AUTHOR]

Year	Author(s)	Title	Journal	Abstract
2022	Ronelle Burger, Timothy Köhler, Aleksandra M. Golos, Alison M. Buttenheim, René English, Michele Tameris and Brendan Maughan-Brown	Longitudinal changes in COVID-19 vaccination intent among South African adults: evidence from the NIDS-CRAM panel survey, February to May 2021	BMC Public Health	Background: COVID-19 vaccine hesitancy has threatened the ability of many countries worldwide to contain the pandemic. Given the severe impact of the pandemic in South Africa and disruptions to the roll-out of the vaccine in early 2021, slower-than-expected uptake is a pressing public health challenge in the country. We examined longitudinal changes in COVID-19 vaccination intent among South African adults, as well as determinants of intent to receive a vaccine. Methods: We used longitudinal data from Wave 4 (February/March 2021) and Wave 5 (April/May 2021) of the National Income Dynamics Study: Coronavirus Rapid Mobile Survey (NIDS-CRAM), a national and broadly representative panel survey of adults in South Africa. We conducted cross-sectional analyses on aggregate and between-group variation in vaccination intent, examined individual-level changes between waves, and modeled demographic predictors of intent. Results: We analysed data for 5629 (Wave 4; 48% male, mean age 41.5 years) and 5862 (Wave 5; 48% male, mean age 41.6 years) respondents. Willingness to get a COVID-19 vaccine significantly increased from 70.8% (95% CI: 68.5–73.1) in Wave 4 to 76.1% (95% CI: 74.2–77.8) in Wave 5. Individual-level analyses indicated that only 6.6% of respondents remained strongly hesitant between survey waves. Although respondents aged 18–24 years were 8.5 percentage points more likely to report hesitancy, hesitant respondents in this group were 5.6 percentage points more likely to change their minds by Wave 5. Concerns about rushed testing and safety of the vaccines were frequent and strongly-held reasons for hesitancy. Conclusions: Willingness to receive a COVID-19 vaccine has increased among adults in South Africa, and those who were entrenched in their reluctance make up a small proportion of the country's population. Younger adults, those in formal housing, and those who trusted COVID-19 information on social media were more likely to be hesitant. Given that stated vaccination intent may not translate into behaviour, our finding that three-quarters of the population were willing to accept the vaccine may reflect an upper bound. Vaccination promotion campaigns should continue to frame vaccine acceptance as the norm and tailor strategies to different demographic groups. [ABSTRACT FROM AUTHOR]

Year	Author(s)	Title	Journal	Abstract
2021	S. A. Bono, E. Faria de Moura Villela, C. S. Siau, W. S. Chen, S. Pengpid, M. T. Hasan, P. Sessou, J. D. Ditekemena, B. O. Amodan, M. C. Hosseinipour, H. Dolo, J. N. Siewe Fodjo, W. Y. Low and R. Colebunders	Factors Affecting COVID-19 Vaccine Acceptance: An International Survey among Low- and Middle-Income Countries	Vaccines	All countries had lower odds for COVID-19 vaccine acceptability compared to Brazil at 90% effectiveness. However, at 95% effectiveness, Thailand (aOR: 1.54, 95% CI [1.14, 2.10], p = 0.006) and Bangladesh (aOR: 1.43, 95% CI [1.08, 1.90], p = 0.012) had higher odds for vaccine acceptability. Compared to participants aged 60 years and above, those in the age groups of 18–29 years and 30–39 years had higher odds of vaccine acceptance at both effectiveness levels, especially among 18- to 29-year-olds at the 95% effectiveness level (aOR: 1.62, 95% CI [1.14, 2.28], p = 0.007). Females had lower odds of willingness to be vaccinated at the 95% effectiveness level (aOR: 0.75, 95% CI [0.65, 0.88], p < 0.001). In terms of income, those with lower-middle (aOR: 1.23, 95% CI [1.01, 1.49], p < 0.001, higher-middle (aOR: 1.75, 95% CI [1.42, 2.16], p < 0.001), and high income (aOR: 1.90, 95% CI [1.32, 2.73], p < 0.001) had higher odds of willingness to be vaccinated compared to those with low income at the 90% effectiveness level.<br>In terms of education and knowledge, participants from undergraduate and postgrad- uate levels had higher odds for willingness to be vaccinated compared to those who had completed primary and secondary education, particularly among undergraduate degree holders at the 95% effectiveness level (aOR: 1.50, 95% CI [1.19, 1.89], p = 0.001). Those who scored higher in COVID-19 knowledge had consistently higher odds of willingness to be vaccinated, particularly at the 95% effectiveness level (aOR: 2.13, 95% CI [1.96, 2.31], p < 0.001).<br>In terms of health status, participants who had tested negative for COVID-19 had higher odds of willingness to be vaccinated both at the 90% effectiveness level (aOR: 1.35, 95% CI [1.19, 1.53], p < 0.001) and at the 95% effectiveness level (aOR: 1.37, 95% [CI 1.15, 1.63], p < 0.001). The presence of at least one underlying chronic disease predicted lower odds for willingness to be vaccinated (aOR: 0.81, 95% CI [0.71, 0.92], p = 0.001) at the 90% effectiveness level. Participants who gave a higher rating to the importance of taking the vaccine to protect themselves had higher odds of taking the vaccine at both levels of effectiveness, particularly at the 95% effectiveness level (aOR: 2.49, 95% CI [2.34, 2.66], p < 0.001). Increased levels of fear/worry about being infected with COVID-19 consistently predicted higher odds of willingness to take the vaccine at 90% (aOR: 1.32, 95% CI [1.25, 1.38], p < 0.001) and 95% effectiveness (aOR: 1.30, 95% CI [1.20, 1.40], p < 0.001).
2021	S. Handebo, M. Wolde, K. Shitu and A. Kassie	Determinant of intention to receive COVID-19 vaccine among school teachers in Gondar City, Northwest Ethiopia	PLoS One	BACKGROUND: Scientists across the world are working on innovating a successful vaccine that will save lives and end COVID-19 pandemic. World Health Organization (WHO) is working to make sure COVID-19 vaccines can be safely delivered to all those who need them. Indeed, the successful deployment and a sufficient uptake of vaccines is equally important. Acceptance and accessibility of such vaccine is a key indicator of vaccination coverage. OBJECTIVE: This study aimed to assess the determinants of intention to receive COVID-19 vaccine among school teachers in Gondar City. METHODS: An institution based cross-sectional study was conducted from December, 2020 to January, 2021. A total of 301 school teachers selected using stratified simple random sampling were included. Descriptive analysis such as medians, means, proportions, standard deviations and frequencies were computed. Linear regression analysis was done to identify factors associated with intention to receive COVID-19 vaccine. A p-value of less than 0.05 was used to declare statistical significance. RESULTS: The median intention to receive COVID-19 vaccine was 3.33 with interquartile range of 2.67-4.0. Of the participants 54.8% had scored above the median of intention to receive COVID-19 vaccine score. 54% variance in intention to receive COVID-19 vaccine was explained by the independent variables. Being affiliated with other category of religion, bachelor degree educational status, perceived susceptibility, perceived benefit, perceived barrier, and cues to action were significantly associated with the intention to receive COVID-19 vaccine. CONCLUSION: The median score of intention to receive COVID-19 vaccine was 3.33. Socio-demographic and health beliefs influenced the intention to receive the COVID-19 vaccine in the study participant. Policy makers and stakeholders should focus on strong health promotion about risks of the pandemic, benefit, safety, and efficacy of vaccination.

Year	Author(s)	Title	Journal	Abstract
2021	Shelton Kanyanda, Yannick Markhof, Philip Wollburg and Alberto Zezza	Acceptance of COVID-19 vaccines in sub-Saharan Africa: evidence from six national phone surveys	BMJ open	
2021	Sohail Agha, Adaobi Chine, Mathias Lalika, Samikshya Pandey, Aparna Seth, Alison Wiyeh, Alyssa Seng, Nandan Rao and Akhtar Badshah	Drivers of COVID-19 Vaccine Uptake amongst Healthcare Workers (HCWs) in Nigeria	Vaccines	This study applied a behavioral lens to understand drivers of COVID-19 vaccination uptake among healthcare workers (HCWs) in Nigeria. The study used data from an online survey of Nigerian HCWs ages 18 and older conducted in July 2021. Multivariate logistic regression analyses were conducted to examine predictors of getting two doses of a COVID-19 vaccine. One-third of HCWs in our sample reported that they had gotten two doses of a COVID-19 vaccine. Motivation and ability were powerful predictors of being fully vaccinated: HCWs with high motivation and high ability had a 15-times higher odds ratio of being fully vaccinated. However, only 27% of HCWs had high motivation and high ability. This was primarily because the ability to get vaccinated was quite low among HCWs: Only 32% of HCWs reported that it was very easy to get a COVID-19 vaccination. By comparison, motivation was relatively high: 69% of HCWs reported that a COVID-19 vaccine was very important for their health. Much of the recent literature coming out of Nigeria and other LMICs focuses on increasing motivation to get a COVID-19 vaccination. Our findings highlight the urgency of making it easier for HCWs to get COVID-19 vaccinations.
2021	Stacey Orangi, Jessie Pinchoff, Daniel Mwanga, Timothy Abuya, Mainga Hamaluba, George Warimwe, Karen Austrian and Edwine Barasa	Assessing the Level and Determinants of COVID-19 Vaccine Confidence in Kenya	Vaccines	The government of Kenya has launched a phased rollout of COVID-19 vaccination. A major barrier is vaccine hesitancy; the refusal or delay of accepting vaccination. This study evaluated the level and determinants of vaccine hesitancy in Kenya. We conducted a cross-sectional study administered through a phone-based survey in February 2021 in four counties of Kenya. Multilevel logistic regression was used to identify individual perceived risks and influences, context-specific factors and vaccine-specific issues associated with COVID-19 vaccine hesitancy. COVID-19 vaccine hesitancy in Kenya was high: 36.5%. Factors associated with vaccine hesitancy included: Rural regions, perceived difficulty in adhering to government regulations on COVID-19 prevention, no perceived COVID-19 infection risk, concerns regarding vaccine safety and effectiveness, and religious and cultural reasons. There is a need for the prioritization of interventions to address vaccine hesitancy and improve vaccine confidence as part of the vaccine roll-out plan. These messaging and/or interventions should be holistic to include the value of other public health measures, be focused and targeted to specific groups, raise awareness on the risks of COVID-19 and effectively communicate the benefits and risks of vaccines.

Year	Author(s)	Title	Journal	Abstract
2022	T. P. Davis, Jr., A. K. Yimam, M. A. Kalam, A. D. Tolossa, R. Kanwagi, S. Bauler, L. Kulathungam and H. Larson	Behavioural Determinants of COVID-19-Vaccine Acceptance in Rural Areas of Six Lower- and Middle-Income Countries	Vaccines (Basel)	Delayed acceptance or refusal of COVID-19 vaccines may increase and prolong the threat to global public health and the economy. Identifying behavioural determinants is considered a critical step in explaining and addressing the barriers of vaccine refusal. This study aimed to identify the behavioural determinants of COVID-19-vaccine acceptance and provide recommendations to design actionable interventions to increase uptake of the COVID-19 vaccine in six lower- and middle-income countries. Taking into consideration the health belief model and the theory of reasoned action, a barrier analysis approach was employed to examine twelve potential behavioural determinants of vaccine acceptance in Bangladesh, India, Myanmar, Kenya, the Democratic Republic of the Congo (DRC), and Tanzania. In all six countries, at least 45 interviews with those who intended to get the vaccine ("Acceptors") and another 45 or more interviews with those who did not ("Non-acceptors") were conducted, totalling 542 interviews. Data analysis was performed to find statistically significant ($p < 0.05$) differences between Acceptors and Non-acceptors of COVID-19 vaccines and to identify which beliefs were most highly associated with acceptance and non-acceptance of vaccination based on the estimated relative risk. The analysis showed that perceived social norms, perceived positive and negative consequences, perceived risk, perceived severity, trust, perceived safety, and expected access to COVID-19 vaccines had the highest associations with COVID-19-vaccine acceptance in Bangladesh, Kenya, Tanzania, and the DRC. Additional behavioural determinants found to be significant in Myanmar and India were perceived self-efficacy, trust in COVID-19 information provided by leaders, perceived divine will, and perceived action efficacy of the COVID-19 vaccines. Many of the determinants were found to be significant, and their level of significance varied from country to country. National and local plans should include messages and activities that address the behavioural determinants found in this study to significantly increase the uptake of COVID-19 vaccines across these countries.
2021	Theophilus Acheampong, Eli A. Akorsikumah, John Osae-Kwapong, Musah Khalid, Alfred Appiah and John H. Amuasi	Examining Vaccine Hesitancy in Sub-Saharan Africa: A Survey of the Knowledge and Attitudes among Adults to Receive COVID-19 Vaccines in Ghana	Vaccines	The impact of COVID-19 vaccination programmes on disease transmission, morbidity and mortality relies heavily on the population's willingness to accept the vaccine. We explore Ghanaian adult citizens' vaccine hesitancy attitudes and identify the likelihood of participation or non-participation in the government's effort to get citizens vaccinated. A fully anonymised cross-sectional online survey of 2345 adult Ghanaians was conducted from 23 to 28 February 2021. Differences in intentions regarding COVID-19 vaccination were explored using Pearson Chi-square tests. Additionally, multinomial logistic regression was used to analyse the factors associated with willingness to receive vaccines. Responses were weighted using the iterative proportional fitting technique to generate a representative sample. About half (51%) of mostly urban adult Ghanaians over 15 years are likely to take the COVID-19 vaccine if made generally available. Almost a fifth (21%) of the respondents were unlikely to take the vaccine, while another 28% were undecided. Additionally, we find differences in vaccine hesitancy among some socio-demographic characteristics such as age, gender, and primary sources of information. Attaining the proverbial 63% to 70% herd immunity threshold in Ghana is only possible if the preventive vaccination programmes are combined with an enhanced and coordinated public education campaign. Such a campaign should focus on promoting the individual and population-level benefits of vaccination and pre-emptive efforts towards addressing misinformation about vaccines.

Year	Author(s)	Title	Journal	Abstract
2021	U. G. Okafor, A. Isah, J. C. Onuh, C. B. Mgbemena and C. M. Ubaka	Community acceptance and willingness to pay for hypothetical COVID-19 vaccines in a developing country: a web-based nationwide study in Nigeria	Pan Afr Med J	INTRODUCTION: some promising COVID-19 vaccines are soon to be available but getting the African community to accept them may be challenging. This study assessed the acceptability and willingness to pay (WTP) for hypothetical COVID-19 vaccines among Nigerians. METHODS: a cross-sectional, web-based study was conducted among the Nigerian populace. A 20-item questionnaire was used to collect responses through Google form which was shared to consenting participants through two social media platforms. Multivariate logistic regression was used to determine the sociodemographic factors that were predictive of respondents´ willingness to accept the COVID-19 vaccines. Statistical significance was set at p<0.05. RESULTS: six hundred and eighty-nine respondents completed the survey, with 50.5% being females. Exactly 43.3% of respondents reported that they would accept a hypothetical vaccine if it is currently available, 62.1% said they would accept it in the future while 71.1% agreed to accept it if recommended by healthcare providers. A third (31.9%) of respondents accepted the vaccine for their self-protection and half of those not accepting it (51.3%) said they did not want to "be used as an experiment". Respondents who were of oldest ages (aOR=0.330, 95% CI: 0.141-0.767, p=0.010), of Christian religion (aOR=3.251, 95% CI: 1.301-8.093, p=0.011), and aware of a possible vaccine being made available (aOR=0.636, 95% CI: 0.440-0.920) were significantly more unwilling to accept the vaccine. The median range of WTP was US$1.2-2.5. CONCLUSION: there is a low acceptance in Nigeria for a COVID-19 vaccine if it was available now, but much higher if it is recommended by a healthcare provider. A high proportion of willing respondents indicated a positive WTP for the vaccine.

Year	Author(s)	Title	Journal	Abstract
2021	Ugochukwu A. Eze, Kingsley I. Ndoh, Babalola A. Ibisola, Chinemerem D. Onwuliri, Adenekan Osiyemi, Nnamdi Ude, Amalachukwu A. Chime, Eric O. Ogbor, Adegboyega O. Alao and Ashiru Abdullahi	Determinants for Acceptance of COVID-19 Vaccine in Nigeria	Cureus	Background: The coronavirus disease 2019 (COVID-19) pandemic heralded an unprecedented race to the development of several vaccine candidates at record speeds never seen in global health. Within nine months, Pfizer-BioNTech's COVID-19 vaccine was approved by the United States FDA. Unfortunately, while these advances were ongoing, there was a burgeoning epidemic of disinformation about the virus and the vaccines that affected the willingness of people, especially minority groups, to get vaccinated. In Nigeria, this wave of vaccine hesitancy was happening against the backdrop of landmark pharmaceutical litigations such as the 2007 Pfizer trovafloxacin lawsuit in the country.; Aim: To assess the determinants of the COVID-19 vaccine's acceptability among Nigerians.; Materials and Methods: Following ethical approval, a population-based cross-sectional study was conducted from November 2020 to January 2021 using an adapted pretested, self-administered questionnaire originally designed by Amyn Malik and colleagues who conducted a similar study at Yale University School of Public Health. The participants were recruited through simple random sampling using a list of community and corporate sites obtained from Google Maps in the three regional zones of Nigeria (north, east, and west) in diverse occupational and residential settings. Information obtained includes socio-demographics, medical history related to COVID-19, level of knowledge, risk perception, and attitudes toward COVID-19 and the vaccines. Descriptive and inferential statistics were done, and results were summarized into percentages and associations. The level of statistical significance was set at a p-value of <0.05. Using the open EpiR package (Emory), we determined a minimum of 340 participants for a statistical power of 80%.; Results: A total of 358 responses were obtained out of the 120 questionnaires distributed in each of the three regions, of which 189 (53%) were females. The mean age of respondents was 32 years (±11.2 SD). About 75% of the participants had at least a college education. The majority (66.2%) of the participants were willing to accept the approved vaccine. The mean risk perception score for COVID-19 was 5.1 (±2.2 SD) out of 10, while the mean COVID-19 symptom knowledge score was 8.6 (±4.1 SD) out of 19. Variables such as being male, identifying as Christian, Hausa ethnicity, and living in northern Nigeria had a statistically significant relationship with the willingness to get vaccinated.; Conclusion: Over 60% of Nigerians are willing to take the COVID-19 vaccines if recommended by health workers. We found male gender, religion, ethnicity, and geographical location to positively influence the willingness of Nigerians to get vaccinated against COVID-19. Health workers should be supported to go beyond the confines of the hospital to educate the general public in schools, marketplaces, churches, and corporate organizations on the efficacy and safety of the approved vaccines. (Copyright © 2021, Eze et al.)

Year	Author(s)	Title	Journal	Abstract
2021	Umakrishnan Kollamparambil, Adeola Oyenubi and Chijioke Nwosu	COVID19 vaccine intentions in South Africa: health communication strategy to address vaccine hesitancy	BMC Public Health	<bold>Background: </bold>Vaccine hesitancy is emerging as a significant challenge in many parts of the world in the fight against the COVID19 pandemic. The continued infection amongst the unvaccinated can lead to a heightened risk of further virus mutation, exposing even those vaccinated to new virus strains. Therefore, there are social benefits in minimising vaccine hesitancy. The objective of this study is to assess the level of COVID19 vaccine hesitancy in South Africa, identify the socio-economic patterns in vaccine hesitancy and highlight insights from the national survey that can inform the development of a COVID-19 vaccination acceptance communication campaign.<bold>Methods: </bold>The study uses the nationally representative National Income Dynamics Study - Coronavirus Rapid Mobile Survey (NIDS-CRAM) survey. The analysis combines univariate and bivariate statistics, as well as multivariate regression models like binomial/ordinal and multinomial logit.<bold>Results: </bold>The study finds that vaccine acceptance is lower than that of non-pharmaceutical intervention like face-mask use. Only 55% fully accept the vaccine, while a further 16% are moderately accepting of vaccines. Together, vaccine acceptance is estimated at 70.8%, and vaccine hesitancy against COVID19 is estimated at 29.2% amongst the adult South African population. The study has identified the perceived risk of infection with the mediating role of efficacy as a key predictor of vaccine intention. Higher awareness of COVID19 related information and higher household income are correlated with lower vaccine hesitancy. The non-black African population group has significantly high vaccine hesitancy compared to black Africans.<bold>Conclusions: </bold>There are other significant differences across socio-economic and demographic variables in vaccine hesitancy. From a communication perspective, it is imperative to continue risk messaging, hand in hand with clearer information on the efficacy of the vaccines. [ABSTRACT FROM AUTHOR]
2021	Yewlsew Fentie Alle and Keder Essa Oumer	Attitude and associated factors of COVID-19 vaccine acceptance among health professionals in Debre Tabor Comprehensive Specialized Hospital, North Central Ethiopia; 2021: cross-sectional study	Virusdisease	
2021	Yitayeh Belsti, Yibeltal Yismaw Gela, Yonas Akalu, Baye Dagnew, Mihret Getnet, Mohammed Abdu Seid, Mengistie Diress, Yigizie Yeshaw and Sofonias Addis Fekadu	Willingness of Ethiopian population to receive COVID-19 vaccine	Journal of Multidisciplinary Healthcare	

Year	Author(s)	Title	Journal	Abstract
2021	Yusuff Adebayo Adebisi, Aishat Jumoke Alaran, Obasanjo Afolabi Bolarinwa, Wuraola Akande Sholabi and Don Eliseo Lucero-Prisno Iii	When it is available, will we take it? Social media users' perception of hypothetical COVID-19 vaccine in Nigeria	Pan African Medical Journal	Introduction: COVID-19 pandemic is a global public health threat facing mankind. There is no specific antiviral treatment for COVID-19, and many vaccine candidates are currently under clinical trials. This study aimed to understand the perception of social media users regarding a hypothetical COVID-19 vaccine in Nigeria. Methods: we conducted a crosssectional survey among social media users in Nigeria in August 2020 using an online questionnaire. The questionnaire includes sections on the demographic characteristics of the respondents and their perception regarding a hypothetical COVID-19 vaccine. A total of 517 respondents completed and returned the informed consent along with the questionnaire electronically. Data were coded and abstracted into Microsoft Excel spreadsheet and loaded into the STATA 14 software for final analysis. Results: the results showed that more than half of the respondents were male 294 (56.9%). Most of the respondents 385 (74.5%) intend to take the COVID-19 vaccine when it becomes available. Among the 132 respondents that would not take the COVID-19 vaccine, the major reason for non-acceptance was unreliability of the clinical trials 49 (37.1%), followed by the belief that their immune system is sufficient to combat the virus 36 (27.3%). We found a significant association between the age of the respondents and the COVID-19 vaccine acceptance (P-value=0.00) as well as geographical location and COVID-19 vaccine acceptance (P-value=0.02). Conclusion: it was observed that most of the respondents were willing to take the COVID-19 vaccine. Our findings also reiterate the need to reassure the public the benefits an effective and safe COVID-19 vaccine can reap for public health. There is a need for national health authorities in Nigeria to ensure public trust is earned and all communities, including the marginalized populations, are properly engaged to ensure an optimal COVID-19 vaccine acceptance. [ABSTRACT FROM AUTHOR]
2021	Z. Iliyasu, A. A. Umar, H. M. Abdullahi, A. A. Kwaku, T. G. Amole, F. I. Tsiga-Ahmed, R. M. Garba, H. M. Salihu and M. H. Aliyu	They have produced a vaccine, but we doubt if COVID-19 exists: correlates of COVID-19 vaccine acceptability among adults in Kano, Nigeria	Hum Vaccin Immunother	Vaccination is a critical tool in the global response to the COVID-19 pandemic. Yet, COVID-19 vaccine hesitancy has not been well explored in parts of Nigeria. We assessed the predictors of acceptability of the COVID-19 vaccine and identified reasons for vaccine hesitancy among adults in urban Kano, northern Nigeria. Using a mixed-methods design, we administered structured questionnaires to a cross-section of adults (n = 446), complemented with 20 in-depth interviews. Binary logistic regression and the framework approach were used to analyze the data. About one-half (51.1%, n = 228) of the respondents were willing to take the COVID-19 vaccine. Vaccine acceptance was higher among older respondents (≥30 years) (adjusted Odds Ratio (aOR) = 1.76, 95% Confidence Interval (CI): 1.14-2.99 (≥30 vs. <30), higher-income earners (≥30,000 Naira) (aOR = 2.06, 95%CI:1.12-3.80, ≥30,000 vs. <30,000), and those with a history of a chronic medical disorder (aOR = 1.90, 95%CI:1.06-3.72). Vaccine acceptance was also higher in persons with high risk perception (aOR = 1.61, 95%CI:1.13-2.81, high vs. low), those who were unconcerned about vaccine safety (aOR = 1.71, 95%CI:1.13-3.55), and those who were not worried about efficacy (aOR = 2.02, 95%CI:1.14-4.11) and infertility-related rumors (aOR = 1.98, 95%CI:1.24-3.18). Themes revealed doubts about the existence of COVID-19, mistrust for authorities, and popular credence to rumors and conspiracy theories. In conclusion, COVID-19 vaccine acceptance was sub-optimal and influenced by respondent's age, income, co-morbidities, risk perception, and concerns about vaccine safety, efficacy, and rumors. Context-specific, evidence-based risk communication strategies and trust-building measures could boost vaccine confidence in similar settings.

Year	Author(s)	Title	Journal	Abstract
2021	Zubairu Iliyasu, Muhammad R. Garba, Auwalu U. Gajida, Taiwo G. Amole, Amina A. Umar, Hadiza M. Abdullahi, Aminatu A. Kwaku, Hamisu M. Salihu and Muktar H. Aliyu	'Why Should I Take the COVID-19 Vaccine after Recovering from the Disease?' A Mixed-methods Study of Correlates of COVID-19 Vaccine Acceptability among Health Workers in Northern Nigeria	Pathogens and global health	We assessed the acceptability of COVID-19 vaccine, predictors, and reasons for vaccine hesitancy among clinical and non-clinical staff at a tertiary hospital in Kano, northern Nigeria.Using a mixed-methods design, structured questionnaires were administered to 284 hospital staff, followed by 20 in-depth interviews with a purposive sub-sample. Logistic regression and the framework approach were used to analyze the data.Only 24.3% (n = 69) of the respondents were willing to accept the COVID-19 vaccine. Acceptance was lower among females (Adjusted Odds Ratio (aOR) = 0.37, 95% Confidence Interval (95%CI): 0.18-0.77 (male vs. female), nurses/midwives (aOR = 0.41, 95%CI:0.13-0.60, physicians vs. nurses/midwives), persons not tested for COVID-19 (aOR = 0.32, 95%CI 0.13-0.79) (no vs. yes) and those who perceived themselves to be at low risk of COVID-19 (aOR = 0.47, 95%CI,0.21-0.89, low vs. high). In contrast, vaccine acceptance was higher among more experienced workers (aOR = 2.28, 95%CI:1.16-8.55, ≥10 vs. <5 years). Vaccine acceptance was also higher among persons who did not worry about vaccine efficacy (aOR = 2.35, 95%CI:1.18-6.54, no vs. yes), or about vaccine safety (aOR = 1.76, 95%CI: 1.16-5.09, no vs. yes), side effects (aOR = 1.85, 95%CI:1.17-5.04, no vs. yes), or rumors (aOR = 2.55, 95%CI:1.25-5.20, no vs. yes). The top four reasons for vaccine hesitancy included distrust, inadequate information, fear of long-term effects, and infertility-related rumors.Concerted efforts are required to build COVID-19 vaccine confidence among health workers in Kano, Nigeria.Our findings can help guide implementation of COVID-19 vaccination in similar settings.
